# Postoperative Pain Management After Lumbar Discectomy. A Systematic Review With Meta‐Analyses and Trial Sequential Analyses

**DOI:** 10.1002/ejp.70261

**Published:** 2026-04-10

**Authors:** Josephine Zachodnik, Rachid Bech‐Azeddine, Magnus Sandberg, Rebecca Scherwin, Rikke Malene Hartvigsen Grønholm Jepsen, Louise Møller Jørgensen, Kasper Højgaard Thybo, Anja Geisler

**Affiliations:** ^1^ Centre for Anaesthesiological Research, Department of Anaesthesiology Zealand University Hospital Koege Denmark; ^2^ Department of Health Sciences, Faculty of Medicine Lund University Lund Sweden; ^3^ Aleris Hospital Copenhagen Denmark; ^4^ Department of Clinical Medicine Copenhagen University Copenhagen Denmark; ^5^ Copenhagen Spine Research Unit (CSRU), Section of Spine Surgery, Center of Rheumatology and Spine Diseases Copenhagen University Hospital Rigshospitalet Denmark; ^6^ Department of Digestive Diseases, Transplantation and General Surgery Copenhagen University Hospital Rigshospitalet Denmark

## Abstract

**Background:**

Inadequate postoperative pain management after lumbar discectomy may delay recovery, increase the risk of chronic pain, and prolong hospitalization. Effective analgesic strategies must balance pain control with minimal adverse effects.

**Objective:**

To identify the most effective postoperative analgesic interventions for patients undergoing lumbar discectomy.

**Databases and Data Treatment:**

This systematic review was preregistered in PROSPERO and conducted in accordance with PRISMA guidelines. Randomized controlled trials were identified through systematic searches in Medline, Embase, and the Cochrane Library. The primary outcome was opioid consumption within 24 h postoperatively. Meta‐analyses were conducted using RevMan, with Trial Sequential Analysis (TSA) to adjust for random errors. Risk of bias was assessed using ROB2, and certainty of evidence was evaluated with GRADE.

**Results:**

A total of 76 RCTs comprising 5617 participants were included, covering 11 analgesic strategies. Paracetamol, NSAIDs, epidural and intrathecal anaesthetics, local infiltration, nerve blocks, gabapentin, and pregabalin significantly reduced 24‐h opioid consumption. Several interventions—including paracetamol, NSAIDs, glucocorticoids, ketamine, epidural and intrathecal anaesthetics, local anaesthetics, nerve blocks, gabapentin, and pregabalin—were also associated with lower pain scores at 6 and 24 h. However, evidence certainty ranged from low to very low due to methodological limitations, small sample sizes, heterogeneity, and inconsistent baseline analgesia.

**Conclusions:**

Multiple analgesic strategies show potential for reducing opioid use and improving early postoperative pain control after lumbar discectomy. Nevertheless, the low certainty of evidence highlights the urgent need for high‐quality, standardized trials to inform clinical practice.

**Significance:**

The findings demonstrate that the following analgesics significantly reduce supplemental opioid consumption and pain levels in the immediate postoperative period: PCM, NSAIDs, intrathecal anaesthetics, epidural anaesthetics, LIA/wound infiltration, nerve blockade, gabapentin, and pregabalin. However, the high risk of bias and low quality of evidence in many of the included trials necessitate cautious interpretation of the findings.

**Abbreviations**
AE: adverse events
CI: confidence interval
DARIS: diversity‐adjusted required information size
Fig: figure
GRADE: grading of recommendation assessment, development, and evaluation
H: hours
IT: intrathecal anaesthetics
LIA: local infiltration anaesthetics
MCID: minimally clinical important difference
NRS: numeric rating scale
NSAID: non‐steroidal anti‐inflammatory drug
PCA: patient‐controlled analgesic
PONV: post‐operative nausea and vomiting
QoL: quality of life
RCT: randomized controlled trial
SAE: serious adverse event
SD: standard deviation
TSA: trial sequential analysis
VAS: visual analog scale

## 1 Introduction

Current data indicate inadequate management of postoperative pain in clinical practice (Gan 2017; Wu and Raja 2024) despite intensified global attention (Gan 2017) and numerous guidelines published by various medical societies (Faculty of Pain Medicine 2020; National Institute of Health 2020; Waelkens et al. 2021).

Adequate pain management after surgery is essential for patients' recovery (Kehlet and Dahl 2003) and is associated with improved outcomes (Devin and McGirt 2015), while inadequate pain management has numerous negative consequences (Dolan et al. 2016; Ishida et al. 2022).

Worldwide, low back pain is a leading contributor to disability, affecting individuals across all age groups and socioeconomic backgrounds (Ferreira et al. 2023; Zhang et al. 2015). Low back pain constitutes one of the most significant disease burdens in the world (Ferreira et al. 2023). Lumbar discectomy, performed nearly 300,000 times annually in the USA (Aljoghaiman et al. 2020), is typically considered only if patients fail to respond to first‐line conservative treatments such as exercise (Ferreira et al. 2023). Managing postoperative pain following lumbar spine surgery presents a distinct challenge (Prabhakar et al. 2022), including increased risk of developing persistent pain (Gerbershagen et al. 2014). Nowadays, a multimodal analgesic approach is recommended after most surgical procedures (Joshi 2023; Kaye et al. 2019), including analgesics with different mechanisms of action combined to obtain a synergistic effect, thereby reducing the need for opioids and the associated side effects (Buvanendran and Kroin 2009; Maheshwari et al. 2020).

The objective of this systematic review was to evaluate the existing literature supporting current evidence on individual analgesics, combination analgesic regimens, and non‐pharmacological pain management strategies administered following lumbar discectomy. A comprehensive search strategy was employed, along with a validated risk of bias tool, measures to minimize random error, and assessment of the overall quality of evidence using the Grading of Recommendations Assessment, Development and Evaluation (GRADE) approach.

## 2 Methods

This systematic review was conducted according to the published protocol (Zachodnik et al. 2022), preregistered with PROSPERO (ID 42020210465), and reported according to the Preferred Reporting Items for Systematic Reviews and Meta‐analyses Statement (Page et al. 2021), following the methodology recommended by the Cochrane Collaboration (Higgins et al. 2021). Trial sequential analysis (TSA) was used in addition to these recommendations (Wetterslev et al. 2017).

### 2.1 Search Strategy, in and Exclusion Criteria

The authors developed the search strategy in collaboration with a librarian specializing in health research. A broad search string, including MeSH and All Fields terms, was designed to include all relevant literature (Appendix S1). The systematic searches were conducted in the following databases: Medline, Embase, and The Cochrane Library. Additionally, reference lists from relevant trials and the first 500 articles in Google Scholar were hand‐searched. The last search was conducted on 30.05.2024.

### 2.2 Eligibility Criteria

All randomized clinical trials (RCTs) (no language restrictions) investigating analgesic interventions compared with placebo, active comparator, or no intervention for adult patients (age ≥ 18 years) undergoing lumbar discectomy were included.

RCTs using a pharmacological/non‐pharmacological approach to reduce postoperative pain, regardless of dosage, single dosage, administration intervals, or duration of treatment reported, were included. The intervention had to be initiated on the day of surgery. Trials with an active treatment as add‐on therapy to any other intervention (i.e., a basic analgesic regimen/standard treatment) were included, but only if the standard treatment was described and delivered similarly in the different intervention groups.

### 2.3 Data Collection Process

Covidence systematic review software (Veritas Health Innovation, Melbourne, Australia) was used to review titles, abstracts, and full texts to assess eligibility according to the predefined inclusion and exclusion criteria. Two authors (the first author (JZ) and one co‐author) evaluated each title and abstract. Reasons for trial exclusion were documented at the full‐text review stage. Data extraction from the included trials was conducted independently by two authors (the first author (JZ) and one co‐author) and then compared in pairs using a standardized extraction sheet.

The corresponding authors were emailed twice to resolve uncertainties or obtain missing data.

### 2.4 Outcomes

The primary outcome was opioid consumption the first 24 h postoperatively. Secondary outcomes were pain levels at rest and during mobilization at 6 ± 2 and 24 ± 4 h postoperatively, postoperative nausea and vomiting (PONV), adverse events (AE), and serious adverse events (SAE) within 24 h postoperatively, persistent pain after 2 months, and health‐related quality of life (HRQOL) after 3 months postoperatively.

### 2.5 Risk of Bias Assessment

All authors assessed the risk of bias for each outcome using the Risk of Bias 2 tool (Higgins et al. 2019).

It was assessed independently and compared in pairs (first author (JZ) and one co‐author). The following five domains were assessed: bias arising from the randomization process, bias due to deviations from intended interventions, bias due to missing outcome data, bias in the measurement of the outcome, and bias in the selection of the reported result. Each domain was judged to be either ‘low risk of bias,’ ‘some concerns,’ or ‘high risk of bias.’ A trial result was judged to be overall high risk of bias if one or more domains were judged to be at ‘high risk’ or ‘some concerns’ and judged to be overall ‘low risk’ of bias if all domains were ‘low risk’ of bias (Higgins et al. 2019).

Disagreements were resolved by discussion until a consensus was reached. If consensus could not be reached or if there were any doubts, the final decision was made by consulting the last author (AG).

### 2.6 Statistical Analyses

The included studies were grouped together based upon the analgesic interventions.

When two or more trials reported the preplanned outcomes, meta‐analyses were conducted according to the recommendations provided by the Cochrane Handbook for Systematic Reviews and Interventions (Higgins et al. 2021) using RevMan version 5.4.1. The intervention's effects were assessed using random and fixed effects chosen after the most conservative point estimate.

We visually inspected forest plots and calculated *I* ^2^ to assess heterogeneity. TSA was performed to control for the risk of type I and type II errors (Wetterslev et al. 2017). To quantify or adjust the observed differences or information size, the diversity‐adjusted required information size (DARIS) was used from the Trial Sequential Analyses (TSA) (Wetterslev et al. 2017). Additionally, forest plots were visually examined to assess the statistical heterogeneity.

TSA was conducted using software developed by the Copenhagen Trial Unit (Wetterslev et al. 2017) for both primary and secondary outcomes. For dichotomous outcomes, we estimated the DARIS based on the observed proportion of participants with an outcome in the control group, assuming a relative risk reduction of 20%, an alpha of 5% for the primary outcome, a beta of 10%, and diversity as suggested by the meta‐analysis. We established a *p*‐value threshold of 0.05 for statistical significance when evaluating the outcomes (Jakobsen et al. 2014).

The GRADE approach, alongside the GRADEpro evaluation tool (GRADEpro Guideline Development Tool; Software), was utilized to assess the certainty of the evidence. The assessment covered five domains: risk of bias, inconsistency, indirectness, imprecision, and publication bias. Lastly, the certainty of the evidence was categorized as high, moderate, low, or very low. The findings were conveyed by following the guidelines of Santesso et al. (Santesso et al. 2024).

### 2.7 Preparation of Data

The dose and route of administration of opioid consumption were converted to intravenous morphine equivalents (Peder et al. 2015). If the trials reported median and interquartile range values, they were directly converted to mean and standard deviation (SD) using the method described by Hozo et al. (2005). Pain scores were extracted if they were convertible to a numeric rating scale (NRS) score. Visual analog scale (VAS) scores were converted to a NRS by dividing by 10.

### 2.8 Assessment of Clinical Significance

A predefined minimal clinical important difference (MCID) of 5 mg was set for cumulative opioid consumption at 24 h postoperatively (Karlsen et al. 2024). This decision was informed by a prior study indicating a morphine‐sparing effect of other non‐opioid analgesics of less than 5 mg/24 h (Myles et al. 2017). Additionally, we chose an MCID for pain scores as a reduction of 10 mm on a VAS, equivalent to 1 point on the NRS (Waelkens et al. 2021).

## 3 Results

**FIGURE 1:**
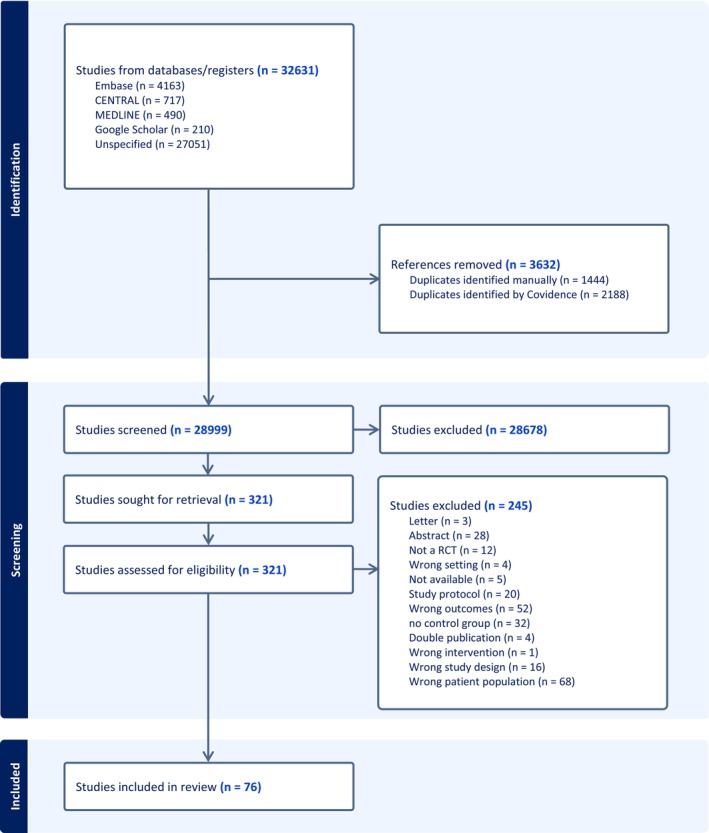
PRISMA flow diagram illustrating the study selection process for inclusion in the systematic review.

From the systematic search, 32.631 trials were identified. After the exclusion of duplicates, 28.999 trials remained. A total of 28.678 trials were excluded during the title and abstract screening. The full texts of 321 trials were retrieved and assessed for eligibility. The main reasons for exclusion were either the incorrect patient population or outcomes. Hence, 76 RCTs matched the inclusion criteria and were included for the final data extraction (Figure 1).

We included 76 clinical trials (Ahn et al. 2003; Altiparmak et al. 2018; Aminmansour et al. 2006; Ammar and Taeimah 2018; Attari et al. 2016; Bahari et al. 2010; Bahçeli and Karabulut 2021; Bilir et al. 2016; Blumenthal et al. 2007; Borjian Boroojeny et al. 2021; Cakan et al. 2008; Ciftci et al. 2020; Cine and Uysal 2023; Demiroglu et al. 2016; Dilmen et al. 2010; El Youssoufi et al. 2001; Elmesallamy and Salem 2024; Fakharian et al. 2009; Farrokhi et al. 2016; Firouzian et al. 2018; Fletcher et al. 1997; Garg et al. 2024; Hadi et al. 2013; Hans et al. 1993; Hegarty and Shorten 2011; Isik et al. 2006; Javery et al. 1996; Karamaz et al. 2004; Kayhan et al. 2019; Kelsaka et al. 2014; Keorochana et al. 2018; Kesimci et al. 2011; Kiabi et al. 2021; Kim et al. 2014; Le Roux and Samudrala 1999; Lotfinia et al. 2007; Mack et al. 2001; Madihalli et al. 2023; Martinez et al. 2013; Mastronardi et al. 2002; Mesut Mese 2023; Milligan et al. 1993; Mirzai et al. 2002; Mitra et al. 2017; Momon et al. 2019; Mondal et al. 2024; Nielsen et al. 2015; Ozmen et al. 2019; Ozyilmaz et al. 2005; Pandey et al. 2004, 2005; Polat et al. 2015; Radhakrishnan et al. 2005; Sahin et al. 2004; Salehpoor et al. 2013; Samoladas et al. 2019; Shimia et al. 2014; Singh et al. 2017; Spreng et al. 2011; SrivaStava et al. 2012; Toygar et al. 2008; Tunali et al. 2013; Tunqkale et al. 2019; Uddin et al. 2022; Uztüre et al. 2020; Uzun et al. 2010; Vahedi et al. 2010, 2011; Wilder‐Smith et al. 1996; Yadav et al. 2018; Yazar et al. 2011; Yazdi et al. 2023; Yörükoǧlu et al. 2005; Youssef and Amin 2016; Zarei et al. 2016), randomizing 5617 participants.

The mean age of included participants was 43, ranging from mean 26–57 years (Kiabi et al. 2021) (Blumenthal et al. 2007), and 43% were female. Of the 76 trials included, 21 provided descriptions of their baseline analgesic regimens, which were heterogeneous and covered various approaches throughout the perioperative period. Notably, multimodal analgesia with paracetamol and nonsteroidal anti‐inflammatory drugs (NSAID) were employed as the baseline regimen in two of these trials (Nielsen et al. 2015; Spreng et al. 2011). For baseline variables of all 76 included trials, see Table 1.

**TABLE 1:** Study information.

Author	Analgesics in intervention and control groups. Type, dose, volume, time points and type of administration	Basic analgesic regimen all groups	Type of supplemental analgesic
Ahn 2003	1: (*n* = 18) Dexamethasone, 5 mg/6 mL NaCl, per operatively, epidural C: (*n* = 18) NaCl, 6 mL, per operatively, epidural	None	PCA Morphine 0.5 mg lockout 8 min
Altiparmark 2018	1: (*n* = 31) Duloxetine, 60 mg, 1 h preoperatively and 24 h postoperatively, pr os. 2: (*n* = 30) Pregabalin, 75 mg, preoperatively, 12 h + 24 h postoperatively C: (*n* = 33) Placebo, 1 caps 1 h preoperatively and 24 h postoperatively, pr os.	End of surgery 1 mg/kg Tramadol iv. × 11 g PCM × 3 POD 0 + POD 1	VAS > 4 Diclofenac 75 mg im
Aminmansour 2006	1: (*n* = 19) Dexamathasone, 40 mgper operatively, iv.2: (*n* = 20) Dexamathasone 80 mg, per operatively, iv. C: (*n* = 22) NaCl, 20 mL per operatively, iv.	None	Morphine to pain relief
Ammar 2018	1: (*n* = 35) Bupivacain 0.25% 20 mL and lidocain 1% 10 mL, per operatively, block C: (*n* = 35) None	PCM 1 g × 4 iv.	PCA Morphine 1 mg iv. lock out 10 min
Attari 2016	1: (*n* = 35) Bupivacain 0.5% 15 mg and Fentanyl 25 μg, per operatively, intratheacal 2: (*n* = 35) Bupivacain 0.5% 15 mg, Fentanyl 25 μg and MgSO_4_ 50% 50 mg, per operatively, intratheacal C: (*n* = 35) Bupivacain, 0.5% 15 mg, per operatively, intratheacal	None	VAS > 3 Morphine 50 μ/kg iv.
Bahari 2010	1: (*n* = 25) Bupivacain 0.5% 1 mL and Adcortyl 10 mg, per operatively, applied topically over the nerve root 2: (*n* = 25) Adcortyl 10 mg and NaCl 1 mL, per operatively, applied topically over the nerve root 3: (*n* = 25) Bupivacain 0.5% 1 mL and NaCl 1 mL, per operatively, applied topically over the nerve root C: (*n* = 25) NaCl 2 mL, per operatively, applied topically over the nerve root	Patients were not limited to any specific pain medication	PCA Opioid
Bahceli 2020	1: (*n* = 47) Progressive relaxation exercise, day 1 + 2 + 3, 30 min × 2 daily C: (*n* = 50) Usual care	NA	NA
Bilir 2016	1: (*n* = 20) Lornoxicam, 8 mg preoperatively and 12 h, postoperatively, iv. 2: (*n* = 20) PCM 1 g preoperatively and 6 + 12 + 18 h, postoperatively, iv. C: (*n* = 20) NaCl, 20 mL preoperatively and 12 h postoperatively, iv.	NA	NRS > 4 PCA Meperidine, 0.5 mg/kg Basal infusion 1 mg, Bolus 5 mg Lock‐out time 10 min
Blumenthal 2007	1: (*n* = 20) Controlled‐release oxycodone, 20 mgpreoperatively and every 12 h, pr os. C: (*n* = 20) Placebo, preoperatively and every 12 h, pr os.	PCM per operatively + every 6 h	PACU:Morphine 2 mg iv. with a delay of 6 min before the next bolus WARD: PCA Morphine 2 mg iv. bolus 8 min lockout time
Boroojeny 2021	1: (*n* = 30) Pregabalin, 75 mgpreoperatively, pr os. 2: (*n* = 30) Pregabalin, 150 mg preoperatively, pr os. C: (*n* = 30) Placebo caps, preoperatively, pr os.	None	VAS > 4 Morfin 2 mg iv.2 mg/h of morphine for up to 12 mg per 4 h of infusion
Cakan 2008	1: (*n* = 20) PCM, 1 g., end of surgery and × 4 daily, iv. C: (*n* = 20) NaCl, 100 mL, end of surgery and × 4 daily, iv.	None	PCA Morphine 1 mg iv lock out 10 min
Ciftci 2020	1: (*n* = 30) Bupivacain 0.25% 40 mL per operatively, ESPB block 2: (*n* = 30) Bupivacain 0.25% 40 mL per operatively, TLIP block C: (*n* = 30) No intervention	PCM iv. 1 g × 4	PCA Fentanyl 2‐mL (10 μg/mL)lock out 20 min VAS > 4 Meperidine 0.5 mg/kg iv. (pacu 24 h)
Cine 2023	1: (*n* = 40) Bupivacain 10 mL 0.5% 50 mg + Methylprednisolon 40 mg 20 min pre incision, epidural 2: (*n* = 40) Bupivacain 10 mL 0.5% 50 mg, 20 min pre incision, epidural C: (*n* = 40) NaCl 20 mL, 20 min pre incision, epidural	NA	Diclofenac sodium Tramadol im
Demiroglu 2016	1: (*n* = 25) Magnesium 50 mg/kg and 150 mL NaCl, per operatively, iv. 2: (*n* = 25) Magnesium 50 mg/kg and 30 mL NaCl, per operatively, im C: (*n* = 25) NaCl 30 mL, im	Tramadol 100 mg iv. per operatively Metoclopramide 10 mg iv. per operatively	PCA Tramadol 16 mg lock out 10 min NRS > 6 Diclofenac 75 mg im
El Youssoufi 2001	1: (*n* = 30) Bupivacain 0.125% 20 mL per operatively, epidural 2: (*n* = 30) Bupivacain 0.125% 19 mL, epidural and Clonindin 150 μg 1 mL, per operatively C: (*n* = 30) NaCl 20 mL, per operatively, epidural	None	VAS > 25 Ketoprofen 100 mg iv. or Proparacetamol 2 g iv.
Esmail 2008	1: (*n* = 83) Lidocaine 0.2% 20 mL per operatively, sc C: (*n* = 83) NaCl 20 mL, per operatively, sc	None	VAS > 3 Morphine 5 mg im
Farrokhi 2016	1: (*n* = 52) Methyl blue 0.5% 1 mL per operatively, epidural C: (*n* = 55) NaCl 1 mL, per operatively, epidural	None	VAS > 6Morphine 4 mg iv.
Firouzian 2018	1: (*n* = 40) Naloxone 0.25 μg/kg/h 21 mL/h, 24 h postoperatively, iv. C: (*n* = 40) NaCl, 21 mL/h, 24 h postoperatively, iv.	PCA morphine 0.5 mg/mL 2 mL/h	PCA Morphine 0.5 mg/mL 0.5 mL lock out 15 min
Fletcher 1997	1: (*n* = 15) Propacetamol 2 g + Gluc ose 125 mL, × 4 daily 48 h, iv. 2: (*n* = 15) Ketoprofen 50 mg + Glucose 125 mL, × 4 daily 48 h, iv. 3: (*n* = 15) Propacetamol 2 g + Ketoprofen 50 mg + Glucose 125 mL × 4 daily 48 h, iv. C: (*n* = 15) Glucose 125 mL × 4 daily 48 h, iv.	None	Postoperatively pain was controlled by a titration of morphine iv. administered by a nurse (3 mg morphine every 10 min until pain VAS score < 30PCA) Morphine 1 mg iv. lock out 10 min
Garg 2024	1: (*n* = 40) Pregabalin 150 mg pr os. + PCM 1 g. iv., preoperatively 2: (*n* = 40) Gabapentin 300 mg pr os. + PCM 1 g. iv., preoperatively C: (*n* = 40) Vitamin B12 2 caps pr os. + PCM 1 g. iv., preoperatively	NA	VAS > 6Tramadol iv.
Hadi 2013	1: (*n* = 15) Ketamine 1 μg/kg/minintraoperatively and NaCl postoperatively, iv. 2: (*n* = 15) Ketamine 1 μg/kg/minintraoperatively and postoperatively, iv. C: (*n* = 15) NaCl, intraoperatively and postoperatively, iv.	None	VAS ≥ 4 PCAMorphine 3 mg iv.
Hans 1993	1: (*n* = 20) Propacetamolper operatively + postoperatively, 2 g × 4, iv. C: (*n* = 20) NaCl, per operatively + postoperatively, iv.	NA	Piritramide 15 mg or 20 mg im
Hegarty 2011	1: (*n* = 14) Pregabalin 300 mgpreoperatively, pr os. C: (*n* = 18) Placebo Sugar capsulespreoperatively, pr os.	Preoperatively: PCM 1 g pr os. +Diclofenac 75 mg iv. Per operatively: Bupivacain 2.5 mg/mL 10 mL woundPostoperatively: PCM 1 g × 4 postoperatively	PCA Morphine 2 mg iv. lock out 6 min
Isik 2005	1: (*n* = 20) Lornoxicam +100 mL NaCl preoperatively, 8 mg, iv. 2: (*n* = 20) Lornoxicam +100 mL NaClpreoperatively, 16 mg, iv. C: (*n* = 20) NaCl 100 mL, preoperatively, iv.	NA	VAS > 4Petidin 1 mg/kg iv.
Iyengar 2023	1: (*n* = 30) Ketamine 0.3 mg/kg iv. bolus + infusion 4 μg/kg/min, per operatively, iv. C: (*n* = 30) NaCl, per operatively, iv.	NA	NRS ≥ 4 Tramadol 50 mg iv.
Javery 1996	1: (*n* = 22) Morphine 1 mg/mL + ketamine 1 mg/mL, 1 mL lock out 6 min, postoperatively, iv. PCA C: (*n* = 20) Morphine 1 mg/mL, 1 mL lock out 6 min, postoperatively, iv. PCA	NA	NA
Kamel 2024	1: (*n* = 36) Bupivacaine 0.25% 15 mL + Dexamethasone 8 mg + Magnesium sulphate 10% 200 mg per operatively, retrolaminar block C: (*n* = 36) None	PCM 15 mg/kg preoperativelyPCM 1 g × 6	VAS > 4 Ketorolac 30 mg iv.
Kararmaz 2004	1: (*n* = 20) Morphine 2 mg Bupivacain 15 mg, Methylprednisolone 80 mg NaCl, per operatively, epidural C: (*n* = 20) NaCl, per operatively, epidural	NA	PCA Morphine 2 mg lock out 10 min VAS > 5 Fentanyl 0.5 μg/kg
Karst 2003	1: (*n* = 17) Celecoxib 200 mg day before surgery, 200 mg 1 h preoperatively 400 mg × 2 daily, pr os. C: (*n* = 17) Placebo, day before surgery, preoperatively, postoperatively × 2 daily pr os.	NA Dexamethasone 20–80 mg, per operatively, depends on the surgeon	PCA Piritramide 2 mg iv. lock out 8 min max 20 mg/4 h
Kayhan 2019	1: (*n* = 15) Morphine 1 mg, per operatively, over dura2: (*n* = 14) Morphine 2 mg, per operatively, over dura C: (*n* = 15) NaCl, per operatively, over dura	NA	PCAMorphine 1 mg iv.lock out 10 minVAS < 4 Morphine 2 mg iv.
Kelsaka 2014	1: (*n* = 25) Dexketoprofen 50 mg + NaCl 2 mL, per operatively, iv. C: (*n* = 25) NaCl 2 mL, per operatively, iv.	NA	PCA Tramadol 20 mg lock out 15 min VAS > 3 Tramadol 1 mg/kg iv.
Keorochana 2018	1: (*n* = 15) Triamcinolone 40 mgepidural, per operatively C: (*n* = 15) NaCl 5 mL, epidural, per operatively	NA	Morphine
Kesimci 2011	1: (*n* = 25) Dexketoprofen 25 mg preoperatively, pr os. 2: (*n* = 25) PCM 500 mg, preoperatively pr os. C: (*n* = 25) Lactose 1 stk, preoperatively, pr os.	NA	PCA Morphine 1 mg lock out 15 min Morphine iv. cont. 0.3 mg/h
Kiabi 2021	1: (*n* = 39) Buprenorphine 2 mg preoperatively, sublingually C: (*n* = 39) Placebo, preoperatively, sublingually	NA	PCA Morphine 25 mg iv. +1 g PCM in NaCl VAS > 5 3 mg morphine increased
Kim 2014	1: (*n* = 24) Lidocain 1.5 mg/kg bolus +2 mg/kg/h, per operatively, iv. C: (*n* = 23) NaCl same amount, per operatively, iv.	NA	PCA Fentanyl infusion 0.1 mg/kg/h (total regimen of 100 mL) PCA Fentanyl infusion 0.1‐mg/kg bolus lockout 15 min + VAS > 3 50 mg Fentanyl iv.
Korkmaz Dilmen2010	1: (*n* = 18) Metamizol 1 mg + 100 mL NaCl, per operatively + postoperatively × 4, iv. 2: (*n* = 20) PCM 1 mg + 100 mL NaClper operatively + postoperatively × 4, iv. 3: (*n* = 20) Lornoxicam 8 mg + 100 mL NaCl, per operatively + postoperatively × 2 + NaCl × 2 C: (*n* = 19) NaCl 100 mL, per operatively + postoperatively × 4	NA	PCA Morphine 1 mg lock out 7 min
Le Roux 1999	1: (*n* = 27) Ketorolac 30 mg per operatively + × 4 36 h, im C: (*n* = 26) None	NA	Morphine 8–15 mg im × 6–8 orMeperidine 75–150 mg im × 6–8 orOxycodone with Acetaminophen pr os. or Codeine and Acetaminophen
Lotfinia 2007	1: (50) Methylprednisolon, 40 mg, per operatively, epidural 2: (50) Bupivacain 0.5% 2 mL, per operatively, epidural C: (50) NaCl, 4 mL, per operatively, epidural	Meperidine 100 mg × 2 im	Na
Mack 2000	1: (*n* = 10) Ketorolac 30 mgper operatively, iv. 2: (*n* = 10) Bupivacain 0.255 15 ccper operatively, wound infiltration C: (*n* = 10) NaCl 15 cc per operatively, wound infiltration	NA	Morphine
Martinez 2013	1: (*n* = 51) Minocycline 100 mg preoperatively + postoperatively, pr os. C: (*n* = 49) Placebo, preoperatively + postoperativelypr os.	PCM 1 g × 4	NRS > 3 Morphine 10 mg pr os. × 6
Mastronadi 2002	1: (*n* = 51) Morphine 1 mg, per operatively wound C: (*n* = 49) NaCl 1 mL, per operatively, wound	NA	Ketorolac 30 mg × 2 pn
Mese 2023	1: (*n* = 34) Peppermint oil 5 drops 2 h postoperatively, inhalation C: (*n* = 34) None	NA	NA
Milligan 1993	1: (*n* = 30) Bupivacain 0.5% 10 mL per operatively, wound C: (*n* = 30) None	NA	PCA Morphine 2 mg lock out 20 min
Mirzai 2002	1: (*n* = 22) Bupivacain 0.25% 20 mL per operatively, wound C: (*n* = 22) NaCl 20 mL per operatively, wound	Methylprednisolon 40 mg, wound per operatively Lidocain 1% 10 mL wound per operatively	Meperimedine 100 mg im
Mitra 2017	1: (*n* = 15) Ropivacain 20 mL + Tramadol 2 mg/kg, per operatively, wound2: (*n* = 15) Ropivacain 20 mL + Dexmedetomidine 2 mL, per operatively, wound C: (*n* = 15) Ropivacain 20 mL per operatively, wound	NA	VAS > 4 Diclofenac 75 mg im
Momon 2019	1: (*n* = 36) Dexamethasone 0.2 mg/kg, per operatively, iv. 2: (*n* = 36) Pregabalin, preoperatively, pr os.3: (*n* = 38) Dexamethasone 0.2 mg/kg per operatively + Pregabalin preoperatively, pr os. C: (*n* = 35) Placbo 1 caps, preoperatively, pr os.	PCM 1 g. + Ketoprofen 1 mg/kg iv. per operatively, 1 g PCM × 4 Ketoprofen 50 mg × 4 + pre incisional infiltration, mixture of 20 mL 0.75% Ropivacaine + 40 mL NaCl and 0.25 mg Epinephrine	NRS > 3 Oxycodon 3 mg iv.every 5 min until NRS < 3+ 5–10 mg pr os. Oxycodon max × 8
Mondal 2024	1: (*n* = 33) Ropivacain 0.25% 20 mL per operatively, TLIF, classic block medial 2: (*n* = 33) Ropivacain 0.25% 20 mL per operatively, TLIF, modified block lateral C: (*n* = 34) None	PCM 15 mg/kg per operatively + ×4	NRS > 3 PCA Fentanyl 20 μg lock out 15 min
Nielsen 2015	1: (*n* = 75) Dexamethason 16 mgper operatively, iv. C: (*n* = 73) NaCl 4 mL, per operatively, iv.	PCM 1 g. × 4NASAID 400 mg × 4	PCA Morphine 2.5 mg iv. lock out 10 minrescue 2.5 mg morphine iv./nurse/pacu
Ozmen 2019	1: (*n* = 40) Bupivacaine 20–40 mL per operatively, blockade C: (*n* = 40) NaCl 6 mL, per operatively, blockade	Dexketoprofen 50 mg over 12 h	PACU: VAS > 4Tramdol 1 mg/kg Ward: PCA Fentanyl 25 μg lock out 15 min
Ozyilmaz 2005	1: (*n* = 20) Lornoxicam 8 mg preoperatively + NaCl 2 mL tissue was closed, iv. 2: (*n* = 20) Lornoxicam 8 mg per operatively + NaCl 2 mL tissue was closed, iv. C: (*n* = 20) NaCl 2 mL, tissue was closed, iv.	NA	PCA Morphine 1 mg lock out 8 min
Pandey 2005	1: (*n* = 20) Gabapentin 300 mg, preoperatively, pr os. 2: (*n* = 20) Gabapentin 600 mg, preoperatively, pr os. 3: (*n* = 20) Gabapentin 900 mg, preoperatively, pr os. 4: (*n* = 20) Gabapentin 1200 mg, preoperatively, pr os. C: (*n* = 20) Placebo 5 caps, preoperatively, pr os.	NA	PCA Fentanyl 1.0 mg/kglock out 10 min
Pandey 2004	1: (*n* = 28) Gabapentin 300 mg, preoperatively, pr os. C: (*n* = 28) Placebo, preoperatively, pr os.	NA	Fentanyl 2 μg/kg
Polat 2015	1: (*n* = 20) PCM 300 mg + Codeine 30 mg, preoperatively, pr os. 2: (*n* = 20) Naproxen 550 mg + codeine 30 mg, preoperatively, pr os. C: (*n* = 20) Placebo, preoperatively, pr os.	NA	PCA Tramadol 20 mg lock out 10 min Vas > 4 Tramadol 1 mg/kg iv.
Radhakrishnan 2005	1: (*n* = 30) Gabapentin 400 mg × 2 preoperatively, pr os. C: (*n* = 30) Placebo 1 caps × 2 preoperatively, pr os.	NA	PCA Morphine 0.02–0.03 mg/kg lock out 10 min
Sahin 2004	1: (*n* = 17) Remifentanil 0.1 μg/kg/min + Ketamin 0.5 mg/kg C: (*n* = 14) NaCl	NA	PCA Morphine bolus 4 mglock out 15 min
Salehpoor 2013	1: (*n* = 40) Morphine 0.1 mg/kg + Dexamethaseone 8 mg, postoperatively, iv. C: (*n* = 40) Morphine 0.1 mg/kgpostoperatively, iv.	NA	Severe pain—Morphine
Samoladas 2019	1: (*n* = 30) Ropivacaine 2% 180 mg + Betamethasone acetate 3 mg, per operatively, epidural C: (*n* = 30) NaCl, per operatively, epidural	PCM 1 g × 3	Tramadol 100 mg
Shimia 2014	1: (*n* = 24) PCM 1 g/100 mL, per operatively, iv. C: (*n* = 28) NaCl 100 mL, per operatively, iv.	NA	Morphine
Singh 2017	1: (*n* = 25) Bupivacain 0.25% bolus 20 mL per operatively +5 mL/h cont. postoperatively, wound 2: (*n* = 25) Bupivacain 0.25% bolus 10 mL per operatively +5 mL/h cont. postoperatively, epidural C: (*n* = 25) %	1 g PCM per operatively	PCA Morphine 1 mg lock out 10 min
Spreng 2011	1: (*n* = 22) Pregabalin 300 mg, preoperatively, pr os. C: (*n* = 24) Placebo 1 caps, preoperatively pr os.	1 g PCM preoperatively + × 3 postoperativelyDiclofenac 50 mg × 3 postoperatively	PCA Morphine 2 mglock out 10 min
Srivastava 2012	1: (*n* = 21) Etoricoxib 120 mg, preoperatively, pr os. C: (*n* = 22) Placebo 1 caps, preoperatively, pr os.	NA	PCA Fentanyl 20 μg lock out 10 min
Toygar 2008	1: (*n* = 30) PCM 1 g, preoperatively + × 4 daily iv. 2: (*n* = 30) PCM 1 g, per operatively + × 4 dailyiv. C: (*n* = 30) %	NA	PCAMorphine 1.5 mglock out 15 min
Tunali 2013	1: (*n* = 18) PCM 1 g/100 mL NaClper operatively + × 4 daily, iv. 2: (*n* = 18) Dexketoprofen 50 mg/100 mL NaCl, per operatively + × 3 daily, iv. C: (*n* = 20) NaCl 100 mL, per operatively + × 3 daily, iv.	NA	PCAMorphine 1 mglock out 7 minVAS > 4 Morphine 2 mg iv.
Tunckale 2019	1: (*n* = 15) Bupivacain 0.5% 20 mL scper operatively 2: (*n* = 15) Bupivacain 0.5% 12 mL sc + 8 mL fascia, per operatively 3: (*n* = 15) Bupivacain 0.5% 10 mL sc + 8 mL fascia +2 mL dura, per operatively C: (*n* = 15) %	NA	VAS > 5 Diclofenac 75 mg im
Uddin 2022	1: (*n* = 35) Lidocain 2% 10 mL, Lia + Triamcinolone 40 mg/mL 2 mL, epidural + Bupivacain 0.5% 3 mL epidural, before closing incision C: (*n* = 35) Lidocain 2% 10 mL, Lia + NaCl 5 mL epidural, before closing incision	1 g PCM iv. × 3	Tramadol 100 iv.
Uztüre 2020	1: (*n* = 30) PCM 1 g × 4 24 h, iv. C: (*n* = 30) %	NA	PCA Tramadol 10 mg lock out 10 min VAS > 3 Tramadol 0.1 kg/kg iv.
Uzun 2010	1: (*n* = 20) PCM 1 g, per operatively, iv. 2: (*n* = 23) PCM 1 g + Metamizole 1 g, per operatively, iv. C: (*n* = 20) no drug	NA	PCA Morphine 1 mg lock out 10 min
Vahedi 2010	1: (*n* = 37) Amitriptyline, 25 mg, preoperatively, pr os. C: (*n* = 40) Placebo 1 caps, preoperatively, pr os.	NA	PCA Morphine bolus 0.1 mg/kg Morphine 0.03 mg/kg lock out 20 min
Vahedi 2011	1: (*n* = 36) Gabapentin, 300 mg, preoperatively, pr os. C: (*n* = 40) Placebo 1 caps, preoperatively, pr os.	NA	PCA Morphine bolus 0.1 mg/kg Morphine 0.03 mg/kg lock out 20 min
Wilder‐Smith 1996	1: (*n* = 15) Fentanyl 3 μg/kg 100 mL NaCl, per operatively, iv. C: (*n* = 15) NaCl 100 mL, per operatively, iv.	NA	PCA Morphine loading bolus 60 μg/kg bolus 25 μg/kg lock‐out 8 min in PACUlock out 15 min on ward background infusion 15 μg/kg during the first 2 h in PACU room
Yadav 2018	1: (*n* = 20) Pregabalin 150 mg, preoperatively, pr os. 2: (*n* = 20) Pregabalin 300 mg, preoperatively, pr os. C: (*n* = 20) B complex, preoperatively, pr os.	NA	PCA Fentanyl Loading 1 μg/kg bolus 0.25–0.5 μg/kg lock out 10 min
Yazar 2011	1: (*n* = 30) Dexketroprofen 50 mg per operatively, postoperatively, iv. C: (*n* = 30) NaCl, per operatively, postoperatively, iv.	NA	PCA Tramadol 25 mg lock out 15 min VAS > 3 Tramadol 25 mg iv.
Yazdi 2023	1: (*n* = 20) Lidocaine 2% 4 mg/kg just before the incisionpercutaneous injection in the muscles surrounding the lumbar discectomy 2: (*n* = 20) Lidocaine 2% 4 mg/kgjust before closing the incisioninjection paraspinalt muscles C: (*n* = 20) None	NA	NA
Yorukoglu 2005	1: (*n* = 20) Morfin 0.1 mg + 1 mL NaCl per operatively, intrathecal 2: (*n* = 20) Morfin 2 mg + 5 mL NaCl per operatively, epidural 3: (*n* = 20) Bupivacain 0.25% 30 mL per operatively, paraspinal muscles and skin C: (*n* = 20) NaCl, per operatively, paraspinal muscles and skin	NA	Meperidine 1 mg/kg im on demand
Youssef 2016	1: (*n* = 15) Gelfoam Methylprednisolone 40 mg per operatively, nerve root/perineural C: (*n* = 15) Gelfoam NaCl, per operatively nerve root/perineural	Diclofenac 75 mg × 2	NA
Zarei 2016	1: (*n* = 35) Pregabalin 300 mg, preoperatively + 150 mg postoperatively × 2 for 14 days, pr os. 2: (*n* = 35) Pregabalin 300 mg preoperatively +150 mg, 12 and 24 h postoperatively + placebo for the next 13 days, pr os. C: (*n* = 35) Placebo, postoperatively 14 dayspr os.	NA	PCA Morphine 2 mg lock out 8 min

Abbreviations: IM = Intramuscular, IV = Intravenous, NA = Not Available, NRS = Numerical Rating Scale, PCA = Patient‐Controlled Analgesic, VAS = Visual Analog Scale.

**FIGURE 2:**
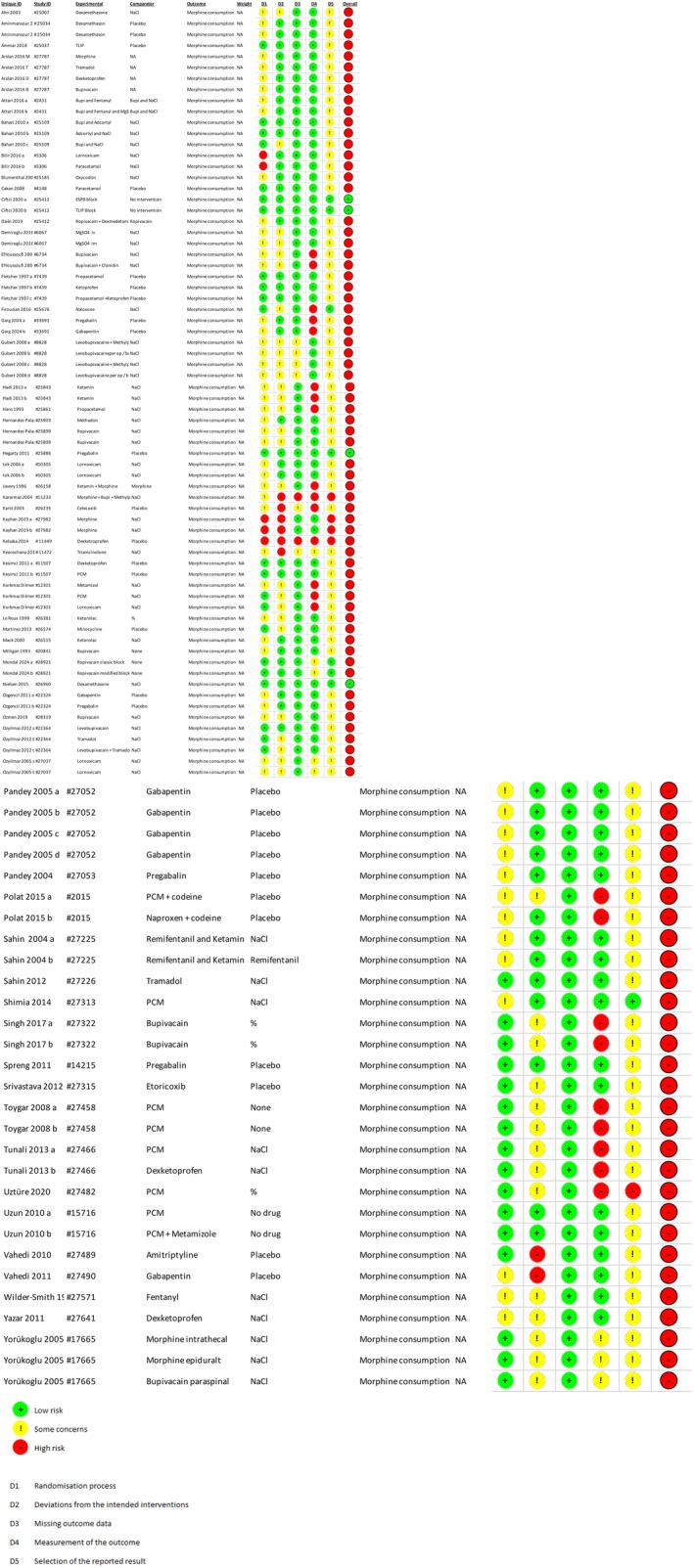
Risk of bias for the primary outcome: Opioid consumption 24 h postoperatively.

When assessing the risk of bias for the primary outcome, 58 of the trials were judged as high risk of bias (Figure 2).

Following data extraction, 11 analgesic groups were identified across 61 of the included trials: opioids, paracetamol, NSAIDs, glucocorticoids, ketamine, epidural anaesthetics, local infiltration anaesthetics (LIA)/wound infiltration, intrathecal anaesthetics (IT), nerve blockades, gabapentin, and pregabalin. Meta‐analytic results and corresponding GRADE assessments of the certainty of evidence for each intervention are summarized in Table 2.

**TABLE 2:** Results.

Outcome/Intervention	Opioid consumption 24 h postoperatively			NRS 6 h postoperatively at rest			NRS 24 h postoperatively at rest			NRS 6 h postoperatively at mobilization			NRS 24 h postoperatively at mobilization			PONV with in 24 h postoperatively	
	Opioid consumption	Point estimate above MCID	GRADE	NRS 6 h	Point estimate above MCID	GRADE	NRS 24 h	Point estimate above MCID	GRADE	NRS 6 h	Point estimate above MCID	GRADE	NRS 24 h	Point estimate above MCID	GRADE	PONV with in 24 h	GRADE
Opioid	NS		Very low	NS		Very low	NS		Very low	NA	NA	NA	NA	NA	NA	NA	NA
Paracetamol	*p* < 0.0001	+	Low	*p* < 0.00001	−	Low	*p* < 0.00001	−	Low	NA	NA	NA	NA	NA	NA	NS	Moderate
NSAIDs	*p* = 0.003	+	Very low	*p* < 0.00001	+	Low	*p* = 0.05	−	Low	NS		Very Low	NS		Very Low	NS	Very low
Glucocorticoid	NA	NA	Moderate	NA	NA	NA	*p* < 0.00001	−	Moderate	NA	NA	NA	*p* = 0.0001	+	Moderate	NS	Moderate
Ketamine	NA	NA	NA	*p* < 0.00001	+	Very low	*p* < 0.00001	+	Very low	NA	NA	NA	NA	NA	NA	*p* = 0.02	Low
Epidural anaesthetics	*p* < 0.0001	+	Low	*p* < 0.00001	+	Low	*p* = 0.02	−	Low	NA	NA	NA	NS		Moderate	NS	Moderate
Intrathecal anaesthetics	*p* < 0.0001	+	Low	*p* = 0.003	+	Very low	NS		Moderate	NA	NA	NA	NA	NA	NA	NS	Very low
LIA/wound infiltration	*p* = 0.04	+	Very low	*p* = 0.001	+	Moderate	*p* = 0.009	−	Moderate	NA	NA	NA	NA	NA	NA	NS	Moderate
Nerve blockade	*p* < 0.0001	+	Low	*p* < 0.00001	+	Low	*p* < 0.0001	−	Moderate	*p* < 0.00001	+	Low	NS		Low	*p* < 0.00001	Low
Gabapentin	*p* = 0.0003	+	Very low	*p* = 0.009	+	Very low	*p* = 0.001	+	Modereate	NA	NA	NA	NA	NA	NA	NS	Very low
Pregabalin	*p* = 0.04	−	Low	*p* = 0.0004	−	Moderate	*p* = 0.006	−	Moderate	NA	NA	NA	NS		Moderate	NS	Low

Abbreviations: h, hours; LIA, Local infiltration anaesthetics; MCID, Minimally Clinical Important Difference; NA, Not available; NRS, Numeric Rating Scale; NS, not significant; NSAIDs, nonsteroidal anti‐inflammatory drugs; PONV, postoperative nausea and vomiting.

Fifteen trials did not meet the criteria for conducting meta‐analyses due to the singularity of the reported intervention. These trials investigated a diverse range of treatments: progressive relaxation exercise (Bahçeli and Karabulut 2021), antidepressant medication (Altiparmak et al. 2018; Uddin et al. 2022) naloxone (Firouzian et al. 2018), antibiotic (Martinez et al. 2013), peppermint oil (Mesut Mese 2023), Magnesium Sulphate (Demiroglu et al. 2016), or various analgesic combinations (Fletcher et al. 1997; Javery et al. 1996; Momon et al. 2019; Polat et al. 2015; Sahin et al. 2004; Salehpoor et al. 2013; Uzun et al. 2010; Vahedi et al. 2010), such as Naproxen combined with Codeine (Polat et al. 2015), Dexamethasone combined with Pregabalin (Momon et al. 2019) or PCM combined with Metamizole (Uzun et al. 2010). The singularity of these interventions prevented their inclusion in the meta‐analysis.

To obtain missing data and uncertainties, 44 corresponding authors were emailed twice, using open‐ended questions to avoid false confirmation of proposed measures. In 17 cases, contact information or the email address was unavailable. Only three authors responded to our inquiries.

**FIGURE 3:**
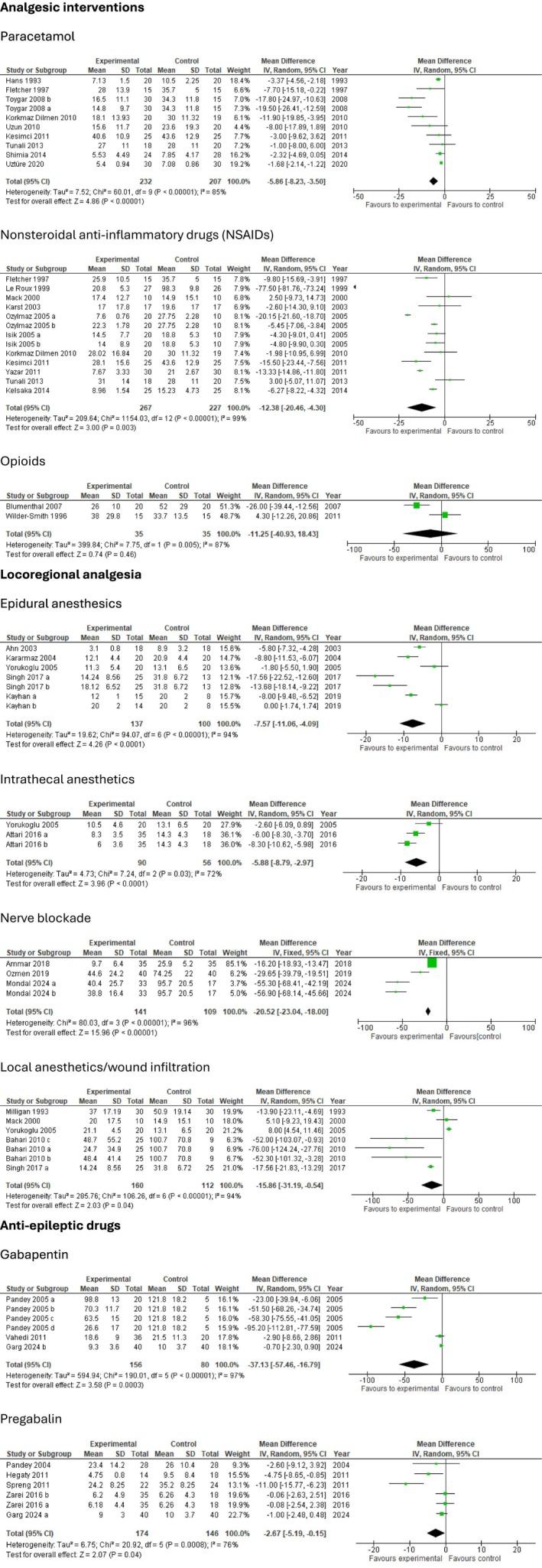
Meta‐analyses for opioid consumption within 24 h postoperatively.

**FIGURE 4:**
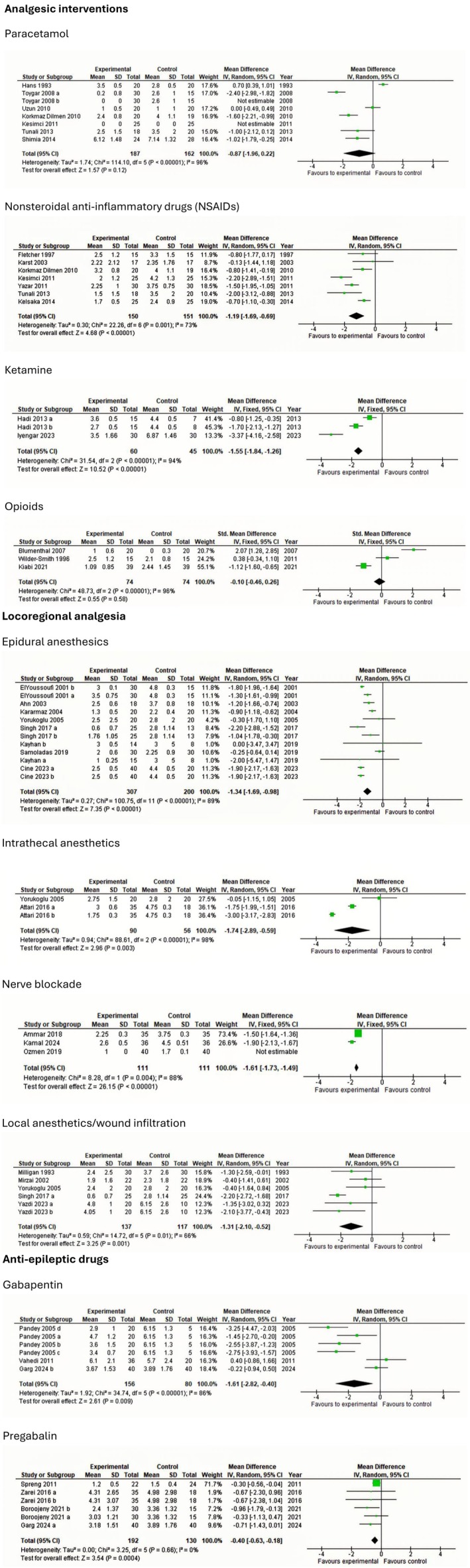
Meta‐analyses for pain at rest 6 h postoperatively.

**FIGURE 5:**
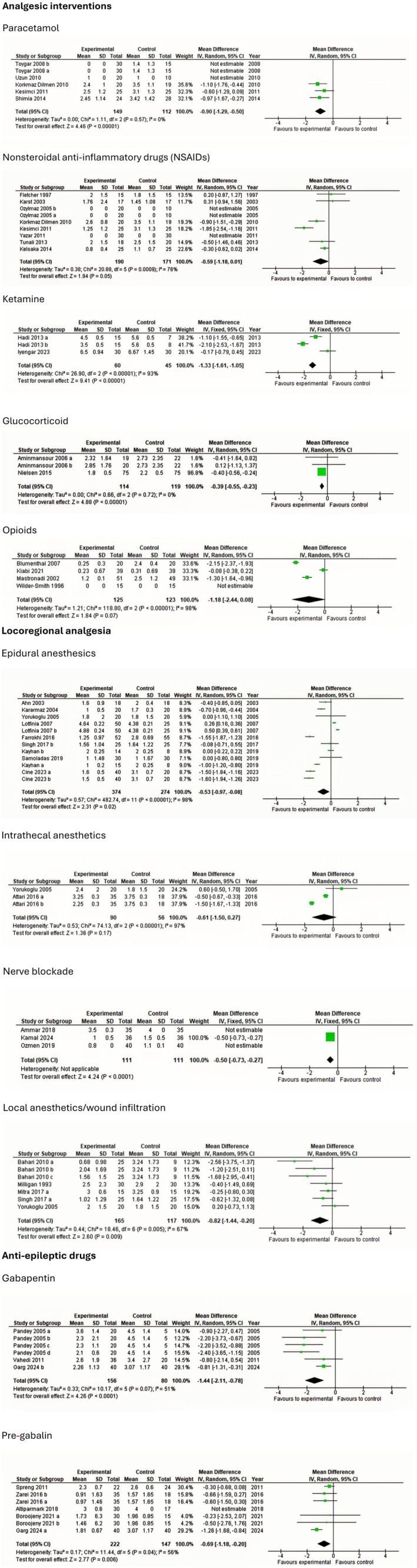
Meta‐analyses for pain at rest 24 h postoperatively.

**FIGURE 6:**
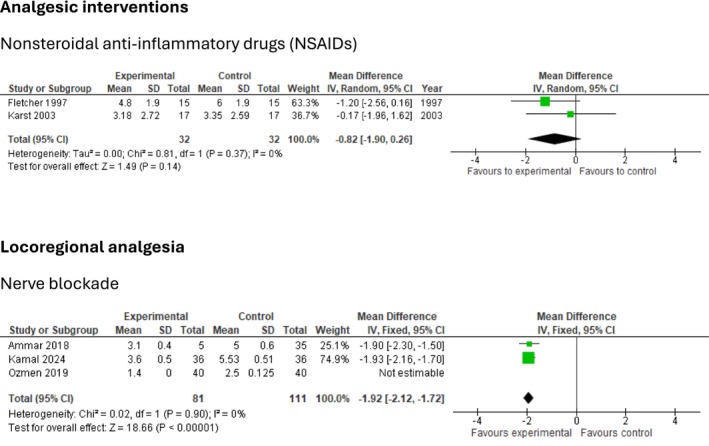
Meta‐analyses for pain during mobilization 6 h postoperatively.

**FIGURE 7:**
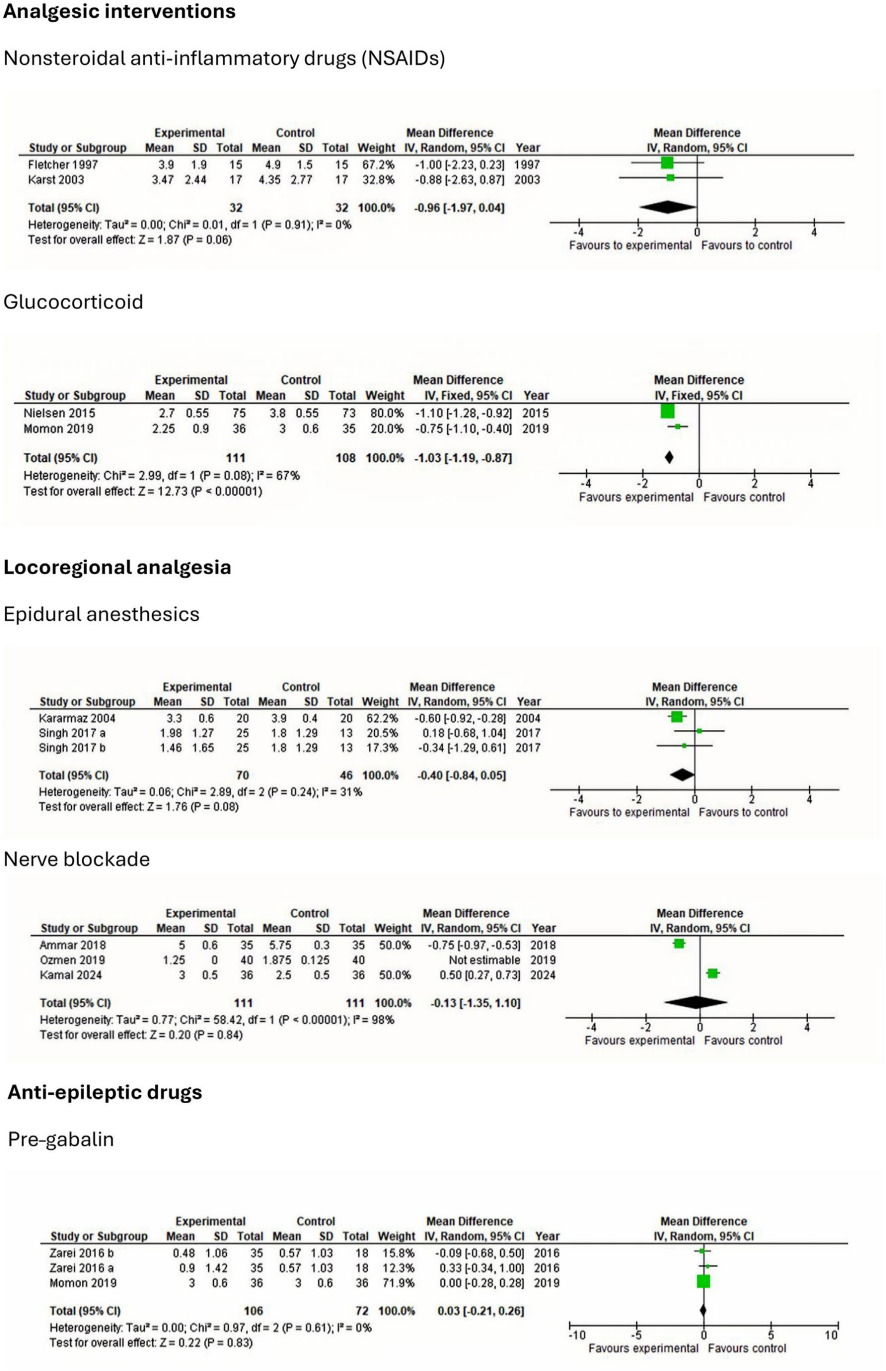
Meta‐analyses for pain during mobilization 24 h postoperatively.

**FIGURE 8:**
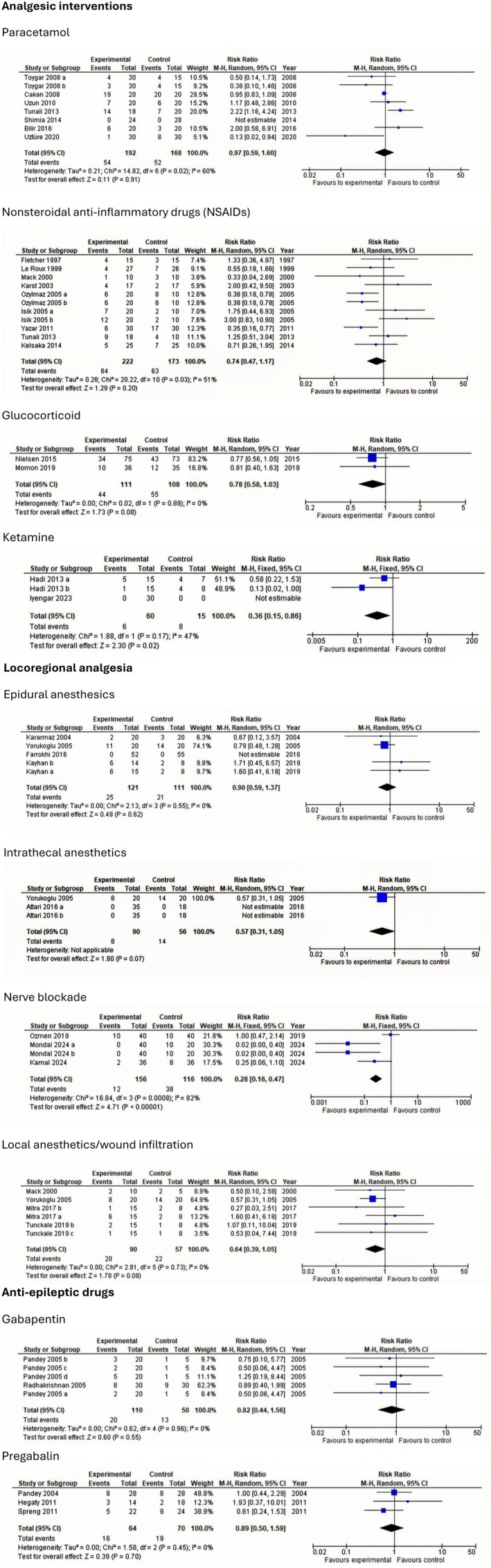
Meta‐analyses for postoperative nausea and vomiting within the first 24 h postoperatively.

The following section presents findings from trials investigating postoperative interventions in patients undergoing lumbar discectomy. To maintain clarity and clinical relevance, only interventions with statistically significant effects on postoperative outcomes are summarized. Meta‐analyses and TSA of non‐significant outcomes are presented in Figures 3, 4, 5, 6, 7, 8.

### 3.1 Group Analysis

#### 3.1.1 Opioid Consumption Within the First 24 h Postoperatively

##### 3.1.1.1 Group Analysis of Analgesic Interventions

###### 3.1.1.1.1 Paracetamol

**FIGURE 9:**
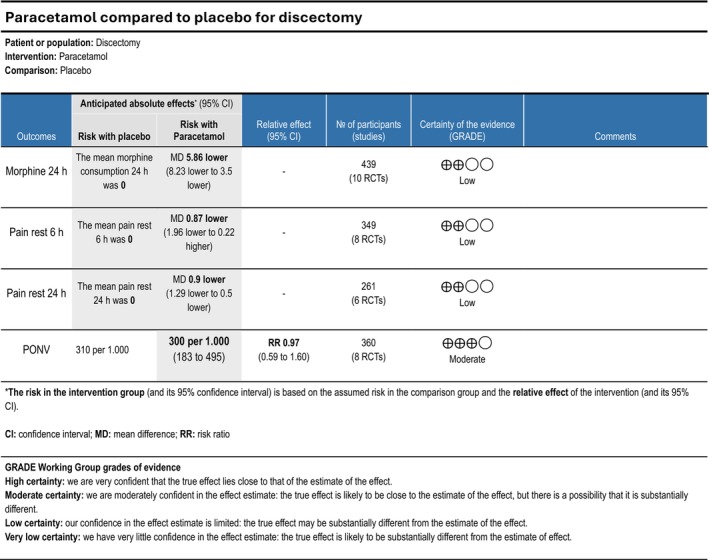
Summarized outcomes in grading of recommendations assessment, development, and evaluation (GRADE) for paracetamol.

Cumulative opioid consumption at 24 h postoperatively was reported in nine trials (Dilmen et al. 2010; Fletcher et al. 1997; Hans et al. 1993; Kesimci et al. 2011; Shimia et al. 2014; Toygar et al. 2008; Tunali et al. 2013; Uztüre et al. 2020; Uzun et al. 2010), including 439 participants. The meta‐analysis demonstrated a statistically significant reduction in opioid consumption compared with control (MD—5.85 mg, 95% CI: −8.23 to −3.5 mg, *p* < 0.05, TSA‐adjusted 95% CI −8.5 to −3.2 mg, DARIS 233, *I* ^2^ = 99%) (Figure 3). The risk of bias for all trials was high, and the quality of evidence (GRADE) was low (Figure 9).

###### 3.1.1.1.2 Nonsteroidal Anti‐Inflammatory Drugs

**FIGURE 10:**
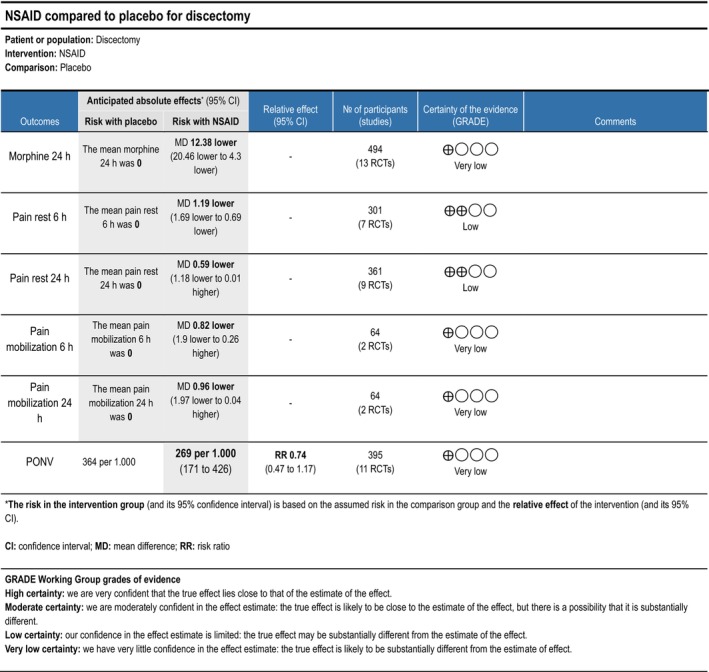
Summarized outcomes in grading of recommendations assessment, development, and evaluation (GRADE) for NSAID.

Cumulative opioid consumption at 24 h postoperatively was reported in 11 trials (Dilmen et al. 2010; Fletcher et al. 1997; Isik et al. 2006; Karst et al. 2003; Kelsaka et al. 2014; Kesimci et al. 2011; Le Roux and Samudrala 1999; Mack et al. 2001; Ozyilmaz et al. 2005; Tunali et al. 2013; Yazar et al. 2011), including 494 participants. The meta‐analysis demonstrated a statistically significant reduction in opioid consumption compared with control (MD—12.38 mg, 95% CI: −20.46 to −4.3 mg, *p* < 0.05, TSA‐adjusted 95% CI: −29.7 to 5.0 mg, DARIS 2299, *I* ^2^ = 99%) (Figure 3). The risk of bias for all trials was high, and the quality of evidence (GRADE) was very low (Figure 10).

##### 3.1.1.2 Group Analysis Anti‐Epileptic Drugs

###### 3.1.1.2.1 Gabapentin

**FIGURE 11:**
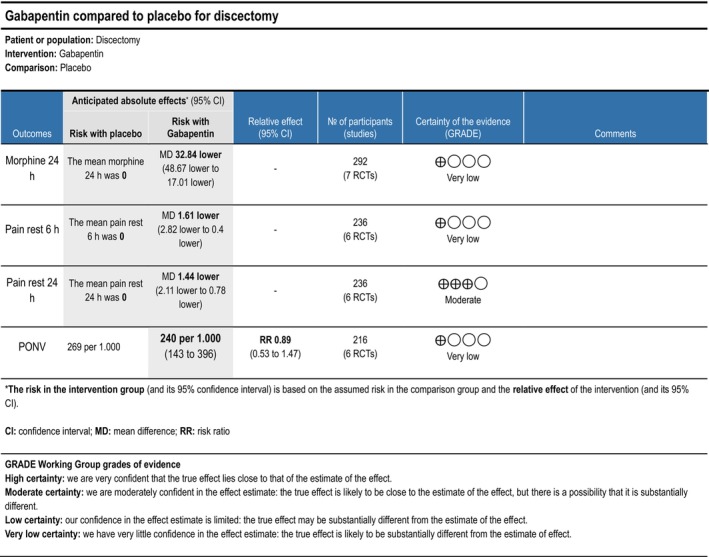
Summarized outcomes in grading of recommendations assessment, development, and evaluation (GRADE) for gabapentin.

Cumulative opioid consumption at 24 h postoperatively was reported in three trials (Garg et al. 2024; Pandey et al. 2005; Vahedi et al. 2011), including 236 participants. The meta‐analysis demonstrated a statistically significant reduction in opioid consumption compared with control (MD—37.13 mg, 95% CI: −57.46 to −16.79 mg, *p* < 0.05, TSA‐adjusted 95% CI −34.5 to 12.5 mg, DARIS 2699, *I* ^2^ = 97%) (Figure 3). The risk of bias for all trials was high, and the quality of evidence (GRADE) was very low (Figure 11).

##### 3.1.1.3 Group Analysis Locoregional Analgesia

###### 3.1.1.3.1 Epidural Anaesthetics

**FIGURE 12:**
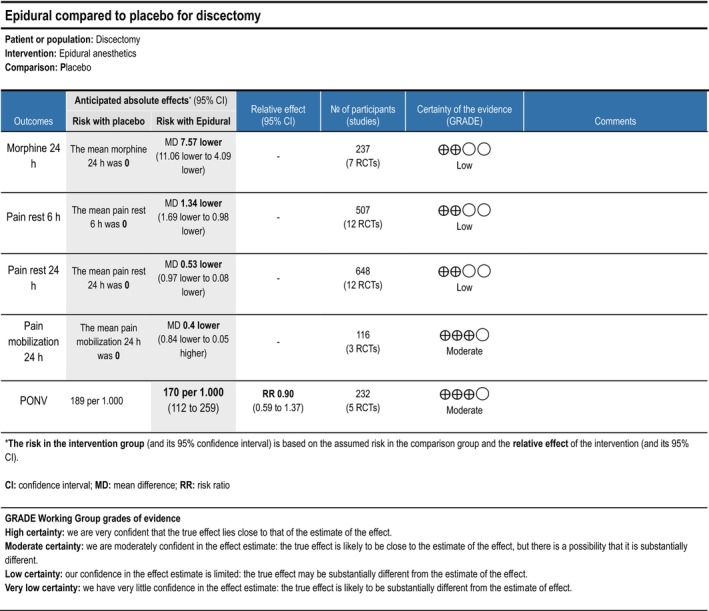
Summarized outcomes in grading of recommendations assessment, development, and evaluation (GRADE) for epidural anaesthetics.

Cumulative opioid consumption at 24 h postoperatively was reported in five trials (Ahn et al. 2003; Al. Kararmaz and Kaya 2004; Kayhan et al. 2019; Singh et al. 2017; Yörükoǧlu et al. 2005), including 237 participants. The meta‐analysis demonstrated a statistically significant reduction in opioid consumption compared with control (MD—7.57 mg, 95% CI: −11.06 to −4.09 mg, *p* < 0.05, TSA‐adjusted 95% CI: −11.5 to −3.7 mg, DARIS 259, *I* ^2^ = 94%) (Figure 3). The risk of bias for all trials was high, and the quality of evidence (GRADE) was low (Figure 12).

###### 3.1.1.3.2 IT Anaesthetics

**FIGURE 13:**
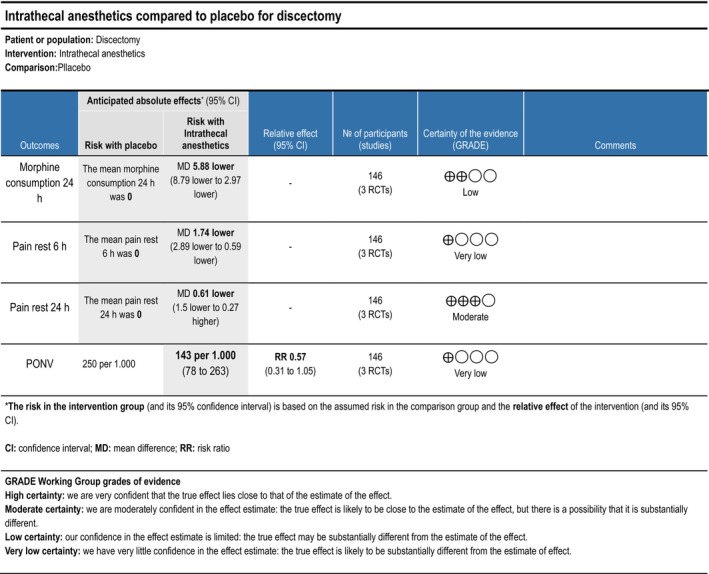
Summarized outcomes in grading of recommendations assessment, development, and evaluation (GRADE) for intrathecal anaesthetic.

Cumulative opioid consumption at 24 h postoperatively was reported in two (Attari et al. 2016; Yörükoǧlu et al. 2005) trials, including 146 participants. The meta‐analysis demonstrated a statistically significant reduction in opioid consumption compared with control (MD—5.88 mg, 95% CI: −8.79 to −2.97 mg, *p* < 0.05, TSA‐adjusted 95% CI: −9 to −2.8 mg, DARIS 103, *I* ^2^ = 72%) (Figure 3). The risk of bias for all trials was high, and the quality of evidence (GRADE) was low (Figure 13).

###### 3.1.1.3.3 Local Anaesthetics/Wound Infiltration

**FIGURE 14:**
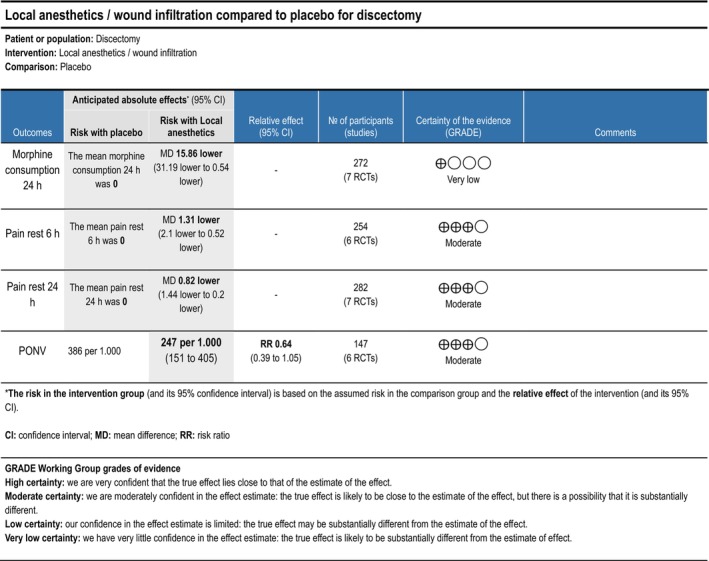
Summarized outcomes in grading of recommendations assessment, development, and evaluation (GRADE) for local anaesthetics/wound infiltration.

Cumulative opioid consumption at 24 h postoperatively was reported in five trials (Bahari et al. 2010; Mack et al. 2001; Milligan et al. 1993; Singh et al. 2017; Yörükoǧlu et al. 2005), including 272 participants. The meta‐analysis demonstrated a statistically significant reduction in opioid consumption compared with control (MD—15.86 mg, 95% CI: −31.19 to −0.54 mg, *p* = 0.04, TSA‐adjusted 95% CI −78.4 to 46.7 mg, DARIS 4766, *I* ^2^ = 94%) (Figure 3). The risk of bias for all trials was high, and the quality of evidence (GRADE) was very low (Figure 14).

###### 3.1.1.3.4 Nerve Blockade

**FIGURE 15:**
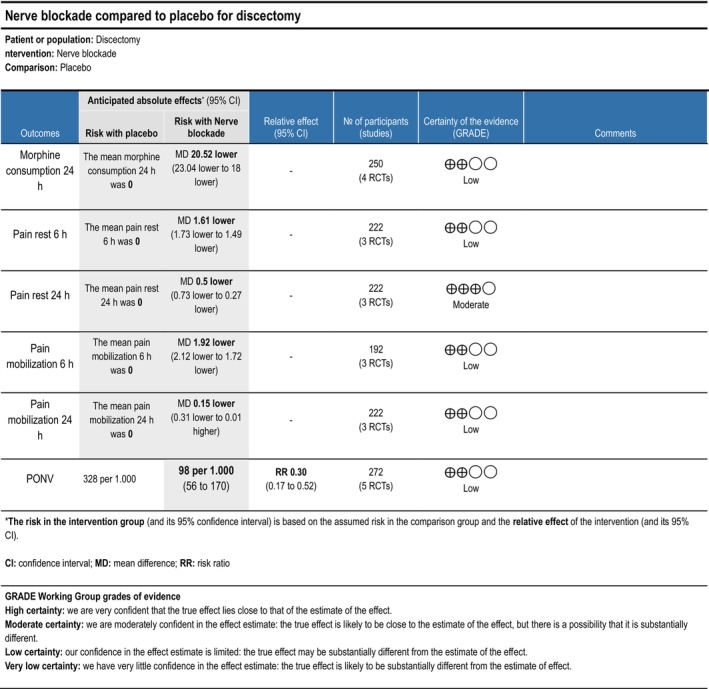
Summarized outcomes in grading of recommendations assessment, development, and evaluation (GRADE) for nerve blockade.

Cumulative opioid consumption at 24 h postoperatively was reported in three trials (Ammar and Taeimah 2018; Mondal et al. 2024; Ozmen et al. 2019), including 250 participants. The meta‐analysis demonstrated a statistically significant reduction in opioid consumption compared with control (MD—20.52 mg, 95% CI: −23.04 to—18.00 mg, *p* < 0.05, *I* ^2^ = 96%, Figure 3). TSA could not be performed due to inadequate information size. The risk of bias for all trials was high, except for one trial where the risk of bias was low. The quality of evidence (GRADE) was low to moderate (Figure 15).

#### 3.1.2 Pain at Rest 6 h Postoperatively

##### 3.1.2.1 Group Analysis of Analgesic Interventions

###### 3.1.2.1.1 Nonsteroidal Anti‐Inflammatory Drugs

Seven trials (Dilmen et al. 2010; Fletcher et al. 1997; Karst et al. 2003; Kelsaka et al. 2014; Kesimci et al. 2011; Tunali et al. 2013; Yazar et al. 2011), including 301 participants, investigated NSAIDs reported postoperative pain at rest after 6 + 2 h. The meta‐analysis demonstrated a statistically significant reduction in NRS compared with control (MD—1.19 NRS, 95% CI: −1.69 to −0.69 NRS, *p* < 0.05, TSA‐adjusted 95% CI: −1.7 to −0.7 NRS, DARIS 154, *I* ^2^ = 73%) (Figure 4). The risk of bias for all trials was high, except for one trial where the risk of bias was some concerns. The quality of evidence (GRADE) was low (Figure 10).

###### 3.1.2.1.2 Ketamine

**FIGURE 16:**
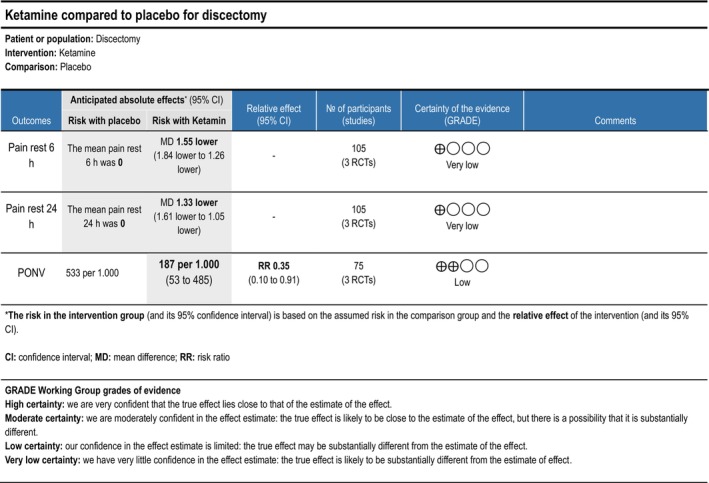
Summarized outcomes in grading of recommendations assessment, development, and evaluation (GRADE) for ketamine.

Two trials (Hadi et al. 2013; Madihalli et al. 2023), including 105, reported ketamine and postoperative pain at rest after 6 + 2 h. The meta‐analysis demonstrated a statistically significant reduction in NRS compared with control (MD—1.55 NRS, 95% CI: −1.84 to −1.26 NRS, *p* < 0.05, TSA‐adjusted 95% CI: −4.1 to 0.3 NRS, DARIS 229, *I* ^2^ = 94%) (Figure 4). The risk of bias for all trials was high, and the quality of evidence (GRADE) was very low (Figure 16).

##### 3.1.2.2 Group Analysis Anti‐Epileptic Drugs

###### 3.1.2.2.1 Gabapentin

Three trials (Garg et al. 2024; Pandey et al. 2005; Vahedi et al. 2011), including 236 participants, investigated gabapentinoids reported postoperative pain at rest after 6 ± 2 h. The meta‐analysis demonstrated a statistically significant reduction in NRS compared with control (MD—1.61 NRS, 95% CI: −2.82 to −0.40 NRS, *p* < 0.05, TSA‐adjusted 95% CI: −3.5 to 0.3 NRS, DARIS 494, *I* ^2^ = 86%). (Figure 4). The risk of bias for all trials was high, and the quality of evidence (GRADE) was very low (Figure 11).

##### 3.1.2.3 Group Analysis Locoregional Analgesia

###### 3.1.2.3.1 Epidural Anaesthetics

Eight trials (Ahn et al. 2003; Cine and Uysal 2023; El Youssoufi et al. 2001; Karamaz et al. 2004; Kayhan et al. 2019; Samoladas et al. 2019; Singh et al. 2017; Yörükoǧlu et al. 2005), including 507 participants, investigating epidural anaesthetics, reported postoperative pain at rest after 6 ± 2 h. The meta‐analysis demonstrated a statistically significant reduction in NRS compared with control (MD—1.34 NRS, 95% CI: −1.69 to −0.98 NRS, *p* < 0.05, TSA‐adjusted 95% CI: −1.7 to −1.0 NRS, DARIS 142, *I* ^2^ = 89%) (Figure 4). The risk of bias for all trials was high, and the quality of evidence (GRADE) was low (Figure 12).

###### 3.1.2.3.2 IT Anaesthetics

Five trials (Milligan et al. 1993; Mirzai et al. 2002; Singh et al. 2017; Yazdi et al. 2023; Yörükoǧlu et al. 2005), including 254 participants, investigating intrathecal anaesthetics reported postoperative pain at rest after 6 + 2 h. The meta‐analysis demonstrated a statistically significant reduction in NRS compared with control (MD—1.31 NRS, 95% CI: −2.10 to −0.52 NRS, *p* < 0.05, TSA‐adjusted 95% CI −2.3 to −0.4 NRS, DARIS 599, *I* ^2^ = 66%) (Figure 4). The risk of bias for all trials was high, and the quality of evidence (GRADE) was very low (Figure 13).

###### 3.1.2.3.3 Local Anaesthetics/Wound Infiltration

Five trials (Milligan et al. 1993; Mirzai et al. 2002; Singh et al. 2017; Yazdi et al. 2023; Yörükoǧlu et al. 2005), including 254 participants, investigating local anaesthetics/wound infiltration reported postoperative pain at rest after 6 + 2 h. The meta‐analysis demonstrated a statistically significant reduction in NRS compared with control (MD—1.31 NRS, 95% CI: −2.10 to −0.52 NRS, *p* < 0.05, TSA‐adjusted 95% CI −2.3 to −0.4 NRS, DARIS 329, *I* ^2^ = 66%) (Figure 4). The risk of bias for all trials was high, and the quality of evidence (GRADE) was moderate (Figure 14).

###### 3.1.2.3.4 Nerve Blockade

Three trials (Ammar and Taeimah 2018; Elmesallamy and Salem 2024; Ozmen et al. 2019), including 222, investigating nerve blockade reported postoperative pain at rest after 6 + 2 h. The meta‐analysis demonstrated a statistically significant reduction in NRS compared with control (MD—1.61 NRS, 95% CI: −1.73 to −1.49 NRS, *p* < 0.05, *I* ^2^ = 88%) (Figure 4). TSA could not be performed due to inadequate information size. The risk of bias was high for one trial and low for two. The quality of evidence (GRADE) was low (Figure 15).

#### 3.1.3 Pain at Rest 24 h Postoperatively

##### 3.1.3.1 Group Analysis of Analgesic Interventions

###### 3.1.3.1.1 Paracetamol

Six trials (Cine and Uysal 2023; Dilmen et al. 2010; Kesimci et al. 2011; Shimia et al. 2014; Toygar et al. 2008; Uzun et al. 2010), including 261 participants investigating paracetamol, reported postoperative pain at rest after 24 ± 2 h. The meta‐analysis demonstrated a statistically significant reduction in NRS compared with control (MD—0.9 NRS, 95% CI: −1.29 to −0.5 NRS, *p* < 0.05, TSA‐adjusted 95% CI −1.3 to 0.5 NRS, DARIS 45, *I* ^2^ = 0%) (Figure 5). The risk of bias for all trials was high, and the quality of evidence (GRADE) was low (Figure 9).

###### 3.1.3.1.2 Nonsteroidal Anti‐Inflammatory Drugs

Eight trials (Dilmen et al. 2010; Fletcher et al. 1997; Karst et al. 2003; Kelsaka et al. 2014; Kesimci et al. 2011; Ozyilmaz et al. 2005; Tunali et al. 2013; Yazar et al. 2011), including 361 participants, investigated NSAIDs reported postoperative pain at rest after 24 + 2 h. The meta‐analysis demonstrated a statistically significant reduction in NRS compared with control (MD—0.54 NRS, 95% CI: −1.18 to 0.01 NRS, *p* = 0.05, TSA‐adjusted 95% CI: −1.3 to 0.1 NRS, DARIS 181, *I* ^2^ = 76%) (Figure 5). The risk of bias for all trials was high, and the quality of evidence (GRADE) was low (Figure 10).

###### 3.1.3.1.3 Glucocorticoid

**FIGURE 17:**
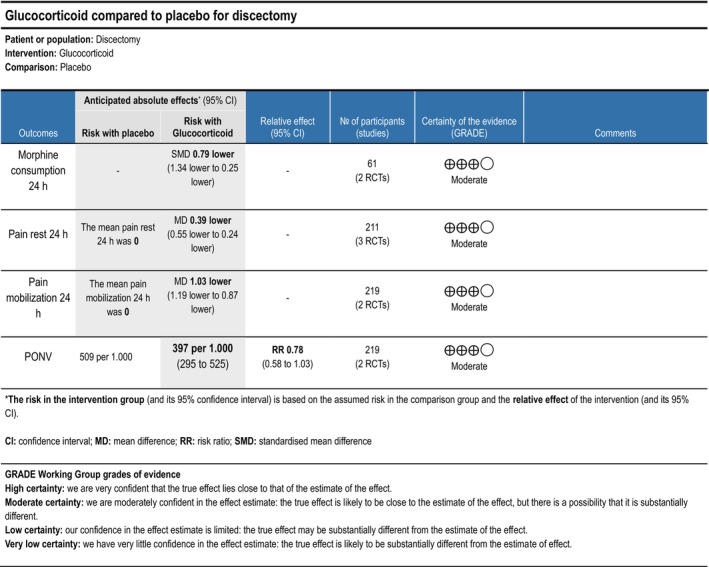
Summarized outcomes in grading of recommendations assessment, development, and evaluation (GRADE) for glucocorticoid.

Two trials (Aminmansour et al. 2006; Nielsen et al. 2015), including 211 participants, investigating glucocorticoids reported postoperative pain at rest after 24 ± 2 h. The meta‐analysis demonstrated a statistically significant reduction in NRS compared with control (MD—0.39 NRS, 95% CI: −0.55 to −0.24 NRS, *p* < 0.05, *I* ^2^ = 0%) (Figure 5). The risk of bias for all trials was high, except for one trial where the risk of bias was low. The quality of evidence (GRADE) was moderate (Figure 17).

##### 3.1.3.2 Group Analysis Anti‐Epileptic Drugs

###### 3.1.3.2.1 Pregabalin

**FIGURE 18:**
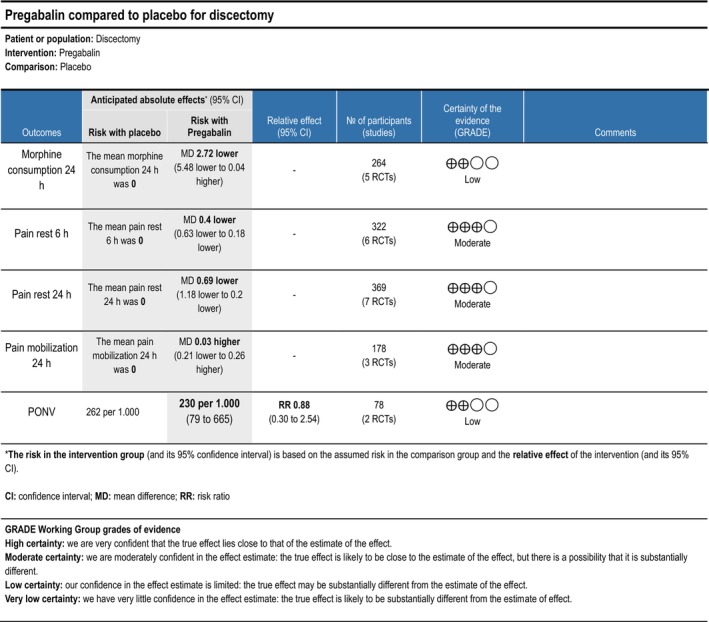
Summarized outcomes in grading of recommendations assessment, development, and evaluation (GRADE) for pregabalin.

Five trials (Altiparmak et al. 2018; Borjian Boroojeny et al. 2021; Garg et al. 2024; Spreng et al. 2011; Zarei et al. 2016), including 369 participants, investigated pregabalin's reported postoperative pain at rest after 24 ± 2 h. The meta‐analysis demonstrated a statistically significant reduction in NRS compared with control (MD—0.69 NRS, 95% CI: −1.18 to −0.20 NRS, *p* < 0.05, TSA‐adjusted 95% CI −1.2 to −0.2 NRS, DARIS 159, *I* ^2^ = 0%) (Figure 5). The risk of bias for all trials was high. The quality of evidence (GRADE) was moderate (Figure 18).

##### 3.1.3.3 Group Analysis Locoregional Analgesia

###### 3.1.3.3.1 Epidural Anaesthetics

Nine trials (Ahn et al. 2003; Farrokhi et al. 2016; Karamaz et al. 2004; Kayhan et al. 2019; Lotfinia et al. 2007; Samoladas et al. 2019; Singh et al. 2017; Yörükoǧlu et al. 2005), including 648 participants investigating epidural anaesthetics, reported postoperative pain at rest after 24 ± 2 h. The meta‐analysis demonstrated a statistically significant reduction in NRS compared with control (MD—0.53 NRS, 95% CI: −0.97 to 0.08 NRS, *p* = 0.02, TSA‐adjusted 95% CI: −1.1 to 0.0 NRS, DARIS 249, *I* ^2^ = 98) (Figure 5). The risk of bias for all trials was high, and the quality of evidence (GRADE) was low (Figure 12).

###### 3.1.3.3.2 Local Anaesthetics/Wound Infiltration

Five trials (Bahari et al. 2010; Milligan et al. 1993; Mitra et al. 2017; Singh et al. 2017; Yörükoǧlu et al. 2005), including 282 participants, investigating local anaesthetics/wound infiltration reported postoperative pain at rest after 24 + 2 h. The meta‐analysis demonstrated a statistically significant reduction in NRS compared with control (MD—0.82 NRS, 95% CI: −1.44 to −0.2 NRS, *p* < 0.05, TSA‐adjusted 95% CI −1.5 to −0.1 NRS, DARIS 221, *I* ^2^ = 67%) (Figure 5). The risk of bias for all trials was high, and the quality of evidence (GRADE) was moderate (Figure 14).

###### 3.1.3.3.3 Nerve Blockade

Three trials (Ammar and Taeimah 2018; Elmesallamy and Salem 2024; Ozmen et al. 2019), including 222 participants, investigating nerve blockade reported postoperative pain at rest after 24 + 2 h. The meta‐analysis demonstrated a statistically significant reduction in NRS compared with control (MD—0.50 NRS, 95% CI: −0.73 to −0.27 NRS, *p* < 0.05). Heterogeneity is not applicable (Figure 5), and TSA could not be performed due to inadequate information size. The risk of bias was high for one trial and low for two. The quality of evidence (GRADE) was moderate (Figure 15).

#### 3.1.4 Pain During Mobilization 6 h Postoperatively

##### 3.1.4.1 Group Analysis Locoregional Analgesia

###### 3.1.4.1.1 Nerve Blockade

Three (Ammar and Taeimah 2018; Elmesallamy and Salem 2024; Ozmen et al. 2019) trials, including 192 participants, investigating nerve blockade reported pain during mobilization after 6 ± 2 h. The meta‐analysis demonstrated a statistically significant reduction in NRS compared with control (MD—1.92 NRS, 95% CI: −2.12 to −1.72 NRS, *p* < 0.05, *I* ^2^ = 0%) (Figure 6). TSA could not be performed due to inadequate information size. The risk of bias was high for one trial and low for two. The quality of evidence (GRADE) was low (Figure 15).

#### 3.1.5 Pain During Mobilization 24 h Postoperatively

##### 3.1.5.1 Group Analysis Analgesic Intervention

###### 3.1.5.1.1 Glucocorticoid

Two trials (Momon et al. 2019; Nielsen et al. 2015), including 219 participants, investigating glucocorticoids reported postoperative pain during mobilization after 24 ± 2 h. The meta‐analysis demonstrated a statistically significant reduction in NRS compared with control (MD—1.03 NRS, 95% CI: −1.19 to −0.87 NRS, *p* < 0.05, *I* ^2^ = 67%) (Figure 7). The risk of bias for all trials was high, except for one trial where the risk of bias was low. The quality of evidence (GRADE) was moderate (Figure 17).

#### 3.1.6 Postoperative Nausea and Vomiting Within the First 24 h

##### 3.1.6.1 Group Analysis of Analgesic Interventions

###### 3.1.6.1.1 Ketamine

*Postoperative nausea and vomiting within the first 24 h postoperatively*: Two trials (Hadi et al. 2013; Madihalli et al. 2023), including 75 participants, investigating ketamine reported on postoperative nausea and vomiting within 24 h. The meta‐analysis demonstrated a statistically significant difference between groups, in favour of the intervention group (RR 0.36, 95% CI: 0.15–0.86, *p* = 0.02, *I* ^2^ = 47%) (Figure 8).

TSA cannot be calculated. The risk of bias for all trials was high, and the quality of evidence (GRADE) was low (Figure 16).

##### 3.1.6.2 Group Analysis Locoregional Analgesia

###### 3.1.6.2.1 Nerve Blockade

Four trials (Ammar and Taeimah 2018; Elmesallamy and Salem 2024; Mondal et al. 2024; Ozmen et al. 2019), including 272 participants, investigated nerve blockade and reported postoperative nausea and vomiting within 24 h. The meta‐analysis demonstrated a statistically significant difference between groups, in favour of the intervention group (RR 0.28, 95% CI: 0.16–0.47, *p* < 0.05, *I* ^2^ = 82%) (Figure 8). TSA could not be performed due to inadequate information size. The risk of bias was high for one trial and low for two. The quality of evidence (GRADE) was low (Figure 15).

#### 3.1.7 Other Outcomes

Meta‐analyses could only be conducted for adverse events (PONV), as very few trials reported other types of AEs and no trials reported SAEs.

One study reported persistent pain 2 months postoperatively (Bahari et al. 2010), and another trial reported quality of life (Farrokhi et al. 2016); meta‐analyses for these outcomes could not be performed.

#### 3.1.8 Qualitative Analyses

The remaining trials reported six interventions, such as progressive relaxation exercise (Bahçeli and Karabulut 2021), antidepressant medication (Altiparmak et al. 2018; Vahedi et al. 2010), naloxone (Firouzian et al. 2018), antibiotic (Martinez et al. 2013), peppermint oil (Mesut Mese 2023), or Magnesium Sulphate (Demiroglu et al. 2016). Furthermore, eight trials (Fletcher et al. 1997; Javery et al. 1996; Momon et al. 2019; Polat et al. 2015; Sahin et al. 2004; Salehpoor et al. 2013; Uzun et al. 2010; Vahedi et al. 2010) investigated different analgesic combinations (for the combinations, please see point three in the results section).

The risk of bias was high in all trials. Six trials demonstrated a statistically significant effect on opioid consumption 24 h postoperatively (Demiroglu et al. 2016; Firouzian et al. 2018; Fletcher et al. 1997; Javery et al. 1996; Martinez et al. 2013; Polat et al. 2015), and seven trials on pain scores (Altiparmak et al. 2018; Firouzian et al. 2018; Javery et al. 1996; Mesut Mese 2023; Polat et al. 2015; Sahin et al. 2004; Vahedi et al. 2010). Two trials demonstrated a statistically significant reduction in opioid‐related adverse events (Demiroglu et al. 2016; Firouzian et al. 2018).

## 4 Discussion

This systematic review assessing pain management after lumbar discectomy identified 11 distinct analgesic categories: opioids, PCM, NSAIDs, glucocorticoids, ketamine, epidural anaesthetics, IT, LIA/wound infiltration, nerve blockades, and antiepileptic drugs. Among these, several interventions statistically significantly impacted opioid consumption after 24 h. The effective interventions included PCM, NSAIDs, epidural anaesthetics, IT, LIA/wound infiltration, nerve blockade, and gabapentin. A similar trend emerged regarding pain at rest, measured at 6 and 24 h postoperatively; NSAIDs, epidural anaesthetics, LIA/wound infiltration, and nerve blockade all showed promising results.

Our findings underscore the limitation that most included trials assessed pain predominantly at rest, with insufficient attention to other key outcomes such as pain during mobilization, persistent pain or chronic pain, AEs, return to daily activities, QoL, and broader recovery trajectories. This finding is also in line with previous research that most pain studies following lumbar discectomy have primarily focused on opioid consumption and pain at rest, while overlooking crucial aspects of postoperative recovery, such as pain during mobilization, QoL, ability to sleep sufficiently, and the development of chronic pain (Beighley et al. 2022). Only a few trials reported patients' QoL postoperatively, which may be considered one of the most crucial outcomes. Furthermore, the lack of QoL was presumably the indication for providing the discectomy in the first place and is therefore vital to monitor postoperatively (Bova et al. 2023). Future research would benefit from adopting a more person‐centred approach, incorporating QoL, the development of persistent pain, and adverse effects preferably as a core outcome set.

At the same time, attention to the overall analgesic strategy remains essential to ensure that pain management supports these broader recovery goals. These findings should be viewed in the context of current recommendations promoting multimodal analgesia after surgical procedures (Joshi 2023; Kaye et al. 2019; Waelkens et al. 2021). Although such an approach aims to enhance analgesic efficacy while minimizing opioid requirements and adverse effects, our review revealed that several included studies did not clearly describe their baseline or multimodal analgesic regimens. This omission makes it challenging to discern whether the reported effects truly reflect the investigated intervention or are influenced by unreported co‐analgesic use. The lack of transparency regarding background analgesia complicates comparison across studies and limits the ability to draw firm conclusions. Hence, future research should ensure detailed reporting of standard perioperative analgesic components to allow valid interpretation of additive effects and to strengthen the evidence base for multimodal postoperative pain management following lumbar discectomy. These reporting gaps reflect a broader methodological challenge in the existing literature, which our review sought to address. Compared with the EJA 2021 review (Waelkens et al. 2021), our analysis adds methodological rigour by applying both the GRADE approach and TSA, highlighting the fragility of the current evidence base. Similar to Geisler et al. (Geisler et al. 2022), our findings emphasize the substantial heterogeneity and inconsistent reporting of analgesic regimens, which continue to obscure the true effectiveness of interventions. These methodological challenges underscore the need for more rigorously designed and transparently reported trials to establish reliable evidence for multimodal pain management following lumbar discectomy.

We acknowledge that many of the included trials are of low quality, which limits the strength and clinical applicability of our conclusions. Consistent with the PROSPECT approach, it is important to consider both the potential benefits and associated risks of the interventions. While several interventions, including multimodal analgesic strategies, may reduce postoperative opioid consumption or pain, their implementation in clinical practice should be guided by careful consideration of both efficacy and safety given the limited high‐quality evidence. In line with the PROSPECT recommendations for spinal surgery (Peene et al. 2021), our findings further underscore the need to balance analgesic efficacy against potential risks when tailoring multimodal analgesia protocols. Incorporating this benefit–risk perspective provides a balanced interpretation and highlights the need for cautious clinical application.

Furthermore, the low certainty of evidence according to the GRADE assessments underscores that the current findings should be interpreted with caution. This fragility limits the strength of clinical recommendations and highlights the need for further high‐quality, adequately powered trials. To strengthen future evidence, research should particularly target areas with the most uncertain evidence, such as specific multimodal analgesic regimens, to establish more robust guidance for postoperative pain management. At the same time, postoperative pain should not be viewed solely through a pharmacological lens. Pain is a multidimensional construct shaped by biological, psychological, and social processes (Engel 2012; Wade and Halligan 2017; Yang et al. 2019). Nonetheless, research and clinical practice remain largely biomedically oriented, often underestimating psychological contributors such as catastrophizing, preoperative anxiety, and patient expectations, which may influence both pain trajectory and postoperative recovery. Given the impact of postoperative pain management on short‐ and long‐term outcomes (Bova et al. 2023), adoption of a fully biopsychosocial framework is imperative for trial design and clinical evaluation.

Building on this perspective, future research should also focus on developing multimodal analgesia protocols specific to lumbar discectomy, similar to those established for other neurosurgical and orthopaedic procedures, to guide high‐quality, methodologically robust trials.

### 4.1 Strengths and Limitations

This systematic review has several methodological strengths, including a comprehensive and methodologically rigorous search strategy that minimizes the chances of overlooking relevant trials, and pre‐registration of the protocol at PROSPERO. TSA was performed to mitigate the risk of random errors and to determine whether the cumulative sample size was sufficient to draw reliable conclusions for each outcome. All included trials underwent a thorough risk of bias assessment using the Cochrane Risk of Bias tool. The certainty of the evidence was systematically evaluated using the GRADE approach, providing a structured basis for understanding the findings.

Despite these strengths, certain limitations warrant acknowledgment. A major concern is the high risk of bias in many of the included trials due to insufficient reporting of critical methodological elements like blinding, randomization, and outcome reporting. The prevalent high risk of bias among trials poses a significant challenge to the reliability of their findings, hampering accurate assessments of treatment efficacy. For several interventions and outcomes, the required information size was not reached, indicating that the evidence remains fragile and that additional high‐quality trials are needed to confirm these findings. Notably, the DARIS threshold was achieved in only three of the assessed outcomes. This indicates that, for the majority of outcomes, the cumulative sample size remains below the required information size. Consequently, further adequately powered and methodologically robust trials are essential to confirm or refute these findings.

Sensitivity analyses based on risk of bias were not feasible, as only a few studies within each intervention were judged to have a low risk of bias. Conducting such analyses with so few trials would not have provided reliable or interpretable results. Future research with more rigorously designed and transparently reported trials is needed to confirm these findings. Consequently, although certain interventions achieved statistical significance, the clinical relevance and generalizability of these effects remain uncertain. Frequent GRADE downgrading was warranted due to methodological concerns, small sample sizes, lack of blinding, and substantial heterogeneity across studies, which challenge the validity of the results. Additionally, baseline analgesic regimens were inconsistently described, making it challenging to attribute effects solely to the investigated interventions. The absence of responses from several authors contacted for quality assessment may have influenced the bias ratings and, subsequently, the GRADE evaluation.

Several interventions achieved reductions in opioid use and pain scores exceeding the designated MCID; however, given the methodological limitations of the included trials, these findings should be interpreted with caution. High‐quality randomized controlled trials with standardized protocols, clearly defined control treatments, and inclusion of patient‐centred outcomes such as pain during mobilization, long‐term quality of life, and persistent pain are urgently needed to establish reliable and clinically meaningful pain management strategies for this surgical population.

## 5 Conclusion

This systematic review offers valuable insights into pain management strategies following discectomy and investigates detailed procedure‐specific pain management following lumbar discectomy. The findings demonstrate that the following analgesics significantly reduce supplemental opioid consumption and pain levels in the immediate postoperative period: Paracetamol, NSAIDs, epidural anaesthetics, LIA/wound infiltration, and nerve blockade. However, due to substantial risk of bias, poor reporting, and low quality of evidence across many included trials, these results should be interpreted cautiously. Importantly, this review does not clarify whether similar benefits can be expected when multiple analgesic techniques are combined. Given the low certainty of evidence and methodological limitations of the included trials, further high‐quality, adequately powered studies are urgently needed to establish robust, procedure‐specific multimodal analgesia protocols.

## Author Contributions

J.Z., M.S., K.H.T., and A.G. – conception and design of the study. J.Z., R.B.‐A., M.S., R.S., R.M.H.G.J., L.M.J., K.H.T., and A.G. – data collection and analysis. J.Z., M.S., K.H.T., and A.G. – drafting and revising the manuscript. J.Z., M.S., K.H.T., and A.G. – interpretation of results. J.Z., R.B.‐A., M.S., R.S., R.M.H.G.J., L.M.J., K.H.T., and A.G. – critical revision and final approval.

## Funding

The authors have nothing to report.

## Conflicts of Interest

The authors declare no conflicts of interest.

## Supporting information


**Appendix S1:** Search strategy.
