# Supplementary material for: Postoperative Pain Management After Lumbar Discectomy. A Systematic Review With Meta‐Analyses and Trial Sequential Analyses
Source: Eur J Pain. 2026 Apr 10;30(4):e70261. doi: 10.1002/ejp.70261 (PMC13067818; doi:10.1002/ejp.70261)
Supplement: Supplementary file 1 — Appendix S1: Search strategy. [file EJP-30-0-s001.pdf]

Appendix 1 Search strategy

Dates: 22-02-2022 and 30-05-2024

Total hits: 32631

Excluded (Duplicates): 3632

Included for primary screening: 28999

Cochrane

All text:

1: *discectomy or diskectomy or discectomies or lumbar discectomy or microdiscectomy or disc surgery or invertebra discectomy or discectomy protusion or disc herniation

Key words: discectomy or diskectomy or discectomies or lumbar discectomy or microdiscectomy or disc surgery or invertebra discectomy or discectomy protusion or disc herniation

2: analgesics or analgesia or opioid or analgesia, opioid or nerve block or local anaesthesics

3: pain or postoperative pain or persistent pain or pain assessment or postoperative pain score

#1 and #2 or #1 and #3

Medline

11: exp Analgesics/ or exp Analgesics, Opioid/ or exp Analgesia/ or exp Nerve Block/ or local anaesthesics.mp.

17: exp Pain/ or exp Pain, Postoperative/ or persistent pain.mp or exp Pain Measurement/ or postoperative pain score.mp

26: exp Diskectomy/ or discektomy.mp. or discectomies.mp or lumbar discectomy.mp or microdiscectomy.mp or disc surgery.mp or discectomy protusion.mp or disc herniation.mp

11 and 26 or 11 and 17

Embase

10: exp discectomy/ or discectomies.mp or lumbar discectomy.mp or exp microdiscectomy/ or diskectomy.mp or disc surgery.mp. or exp intervertebral disk/ or exp intervertebral disk hernia/ or discectomy protusion.mp. or disc herniation.mp

16: analgisics.mp or analgesics opioid.mp or exp analgesia/ or exp nerve block/ or local anaesthesics.mp

23: exp pain/ or exp postoperative pain/ or persistent pain.mp. or exp pain assessment/ or exp pain measurement/ or postoperative pain score.mp.

10 and 16 or 10 and 23

Google scolar

Pain and analgesics and discectomy
